# Melanoma bone metastasis-induced osteocyte ferroptosis via the HIF1α-HMOX1 axis

**DOI:** 10.1038/s41413-024-00384-y

**Published:** 2025-01-16

**Authors:** Yewei Jia, Rui Li, Yixuan Li, Katerina Kachler, Xianyi Meng, Andreas Gießl, Yi Qin, Fulin Zhang, Ning Liu, Darja Andreev, Georg Schett, Aline Bozec

**Affiliations:** 1 Department of Internal Medicine 3, Friedrich-Alexander-University Erlangen-Nürnberg (FAU) and Universitätsklinikum Erlangen, Erlangen, Germany; 2 Deutsches Zentrum Immuntherapie (DZI), Friedrich-Alexander-University Erlangen-Nürnberg (FAU) and Universitätsklinikum Erlangen, Erlangen, Germany; 3 Department of Rheumatology, Renji Hospital, Shanghai Jiao Tong University School of Medicine, Shanghai, China; 4 Department of Opthalmology, Friedrich-Alexander University (FAU) Erlangen-Nürnberg and Universitätsklinikum Erlangen, Erlangen, Germany; 5 Technische Universität Dresden (TUD), Center for Molecular and Cellular Bioengineering (CMCB), Center for Regenerative Therapies Dresden (CRTD), Dresden, Germany

**Keywords:** Bone, Bone cancer

## Abstract

Osteocytes are the main cells in mineralized bone tissue. Elevated osteocyte apoptosis has been observed in lytic bone lesions of patients with multiple myeloma. However, their precise contribution to bone metastasis remains unclear. Here, we investigated the pathogenic mechanisms driving melanoma-induced osteocyte death. Both in vivo models and in vitro assays were combined with untargeted RNA sequencing approaches to explore the pathways governing melanoma-induced osteocyte death. We could show that ferroptosis is the primary mechanism behind osteocyte death in the context of melanoma bone metastasis. HMOX1 was identified as a crucial regulatory factor in this process, directly involved in inducing ferroptosis and affecting osteocyte viability. We uncover a non-canonical pathway that involves excessive autophagy-mediated ferritin degradation, highlighting the complex relationship between autophagy and ferroptosis in melanoma-induced osteocyte death. In addition, HIF1α pathway was shown as an upstream regulator, providing a potential target for modulating HMOX1 expression and influencing autophagy-dependent ferroptosis. In conclusion, our study provides insight into the pathogenic mechanisms of osteocyte death induced by melanoma bone metastasis, with a specific focus on ferroptosis and its regulation. This would enhance our comprehension of melanoma-induced osteocyte death.

## Introduction

The incidence of cutaneous melanoma (CM) has notably increased in developed countries.^1^ Nowadays, around 1.7% of new tumor cases are melanoma cases.^2^ The progression to metastasis significantly impacts morbidity and mortality, even in cases with thin primary tumors.^3^ Among metastatic sites, bone metastases, are observed in 40% of patients, and are associated with a median survival of 2.4 months and a 1-year survival rate of only 10%.^4,5^ In addition to the low survival rates, bone metastases can severely compromise bone integrity, leading to an increased risk of fractures and structural instability.^6,7^ This can often be accompanied by intense pain, which significantly affects the patient’s quality of life.^4^

Osteocytes are the main cells in mineralized bone tissue and played a crucial role in regulating bone metabolism and maintaining bone quality.^8,9^ Although they are prevalent in the bone matrix and have a significant impact on osteoblast and osteoclast activities,^10,11^ their precise contribution to bone metastasis remains unclear. Pathological conditions such as osteoporosis and osteoarthritis are closely associated with incidents of osteocyte death.^12,13^ Furthermore, elevated osteocyte apoptosis has been observed in lytic bone lesions of patients with multiple myeloma.^14^ Although there are indications that melanoma metastasis disrupts the osteocyte network,^15^ the direct mechanisms responsible for melanoma-induced osteocyte death are still unclear.

Recent advances in cell death research have revealed diverse pathways through which cells die. Novel forms of cell death, notably ferroptosis, have been identified.^16–20^ Unlike traditional forms of cell death, ferroptosis is characterized by uncontrolled iron-dependent lipid peroxidation, resulting in specific biological markers such as increased iron accumulation, elevated lipid peroxide levels, and reduced expression of glutathione peroxidase 4 (GPX4). Research targeting ferroptosis has shown potential in managing conditions such as diabetic osteoporosis.^21^ Therefore, we hypothesized that ferroptosis could be responsible as a programmed cell death pathway for osteocyte death caused by melanoma metastasis, with the goal of discovering potential therapeutic approaches.

An intracardiac melanoma metastasis mouse model was used to investigate the influence of ferroptosis on osteocyte demise induced by melanoma metastasis. Both in vivo and in vitro assays demonstrated the important role of ferroptosis in driving this phenomenon. Mechanistically, the increased Hmox1 expression triggered excessive autophagy, resulting in ferritin degradation, subsequent intracellular iron overload, and the onset of lipid peroxidation. Targeting Hmox1 therapeutically efficiently attenuated osteocyte death and preserved trabecular integrity. The regulation of Hmox1 expression was mediated by the upstream transcription factor Hif1α. These findings shed light on the intricate mechanisms underlying melanoma bone metastasis and osteocyte survival.

## Results

### Melanoma bone metastasis induces osteocyte ferroptosis in vivo

We used an in vivo melanoma bone metastasis model by injecting B16F10 melanoma cells into the heart ventricle of mice, simulating melanoma metastasis (Fig. S1A). In previous research, a murine melanoma metastasis model was established with a range of 14 to 28 days.^22,23^ Accordingly, a long-term preliminary experiment was conducted to identify the optimal melanoma injection model. The data demonstrated that mice injected with B16F10 cells exhibited a markedly reduced survival rate after 14 days (Fig. S1B). The mice began to die on day 15 and by day 18, the majority had perished. Therefore, 14 days post tumor injection is an appropriate duration for melanoma metastasis model in subsequent experiments. Body weight was measured at baseline and on day 14, and showed a significant decrease in melanoma metastatic mice compared to the control group (Fig. S1C). H&E staining was then performed to confirm the rate of melanoma bone metastasis. The data showed successful metastasis of melanoma cells into the bone marrow niche after the 14-days metastasis period (Fig. S1D). Subsequent micro-CT analysis revealed a significant reduction in the trabecular bone volume per total volume (Tb.BV/TV), trabecular number (Tb.N), and connectivity density (Conn.D), along with an increase in the trabecular separation (Tb.Sp) in the melanoma group (Fig. S1E). Furthermore, a reduction in cortical bone volume per total volume (Ct.BV/TV) and cortical thickness (Ct.Th) was observed, accompanied by an increase in cortical separation (Ct.Sp) in the cortical bone (Fig. S1F). These data indicate a bone loss phenotype in the melanoma group.

**Fig. 1:**
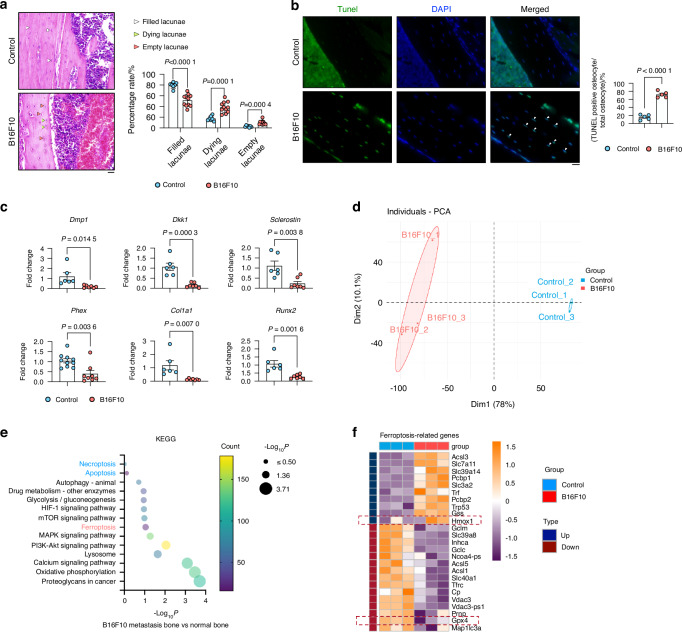
B16F10 Cell-Induced Osteocyte Ferroptosis in Bone Metastasis. **a** H&E staining of cortical bone from control and B16F10-injected mice (*n* ≥ 6). White arrows indicate normal osteocytes; green arrows, dying osteocytes; and red arrows, dead osteocytes. Scale bars: 20 μm. **b** TUNEL staining of tibial bone sections from control and B16F10-injected mice (*n* = 5). White arrows mark TUNEL-positive cells. Scale bars: 20 μm. **c** mRNA expression of *Dmp1, Dkk1, Phex, Sclerostin, Col1a1*, and *Runx2* in long bone (without bone marrow) from control and B16F10-injected mice (*n* ≥ 6). **d** PCA plot showing sample patterns of individual samples in the control and B16F10 groups. **e** KEGG pathway analysis indicating “Ferroptosis” as the significantly altered pathway. **f** Heatmap of differentially expressed genes in ferroptosis pathways. Statistical significance was determined by a 2-tailed Student’s t-test (A, B, C)

We then investigated whether bone metastasis leads to osteocyte death using histochemical analysis and TUNEL staining. Figure 1a shows a higher percentage of empty and dying lacunae (representing dead or dying osteocytes), while the percentage of filled lacunae (representing living osteocytes) was lower in mice injected with melanoma cells, confirming the impact of melanoma metastasis on osteocyte viability. This correlated with the TUNEL assay, which showed a higher number of TUNEL-positive cells in the melanoma group compared to the control group (Fig. 1b).

To further validate changes in osteocyte function following melanoma metastasis, we quantified osteocyte marker genes, namely *Dmp1* (dentin matrix acidic phosphoprotein), *Dkk1* (Dickkopf WNT Signaling Pathway Inhibitor 1), *Phex* (phosphate-regulating endopeptidase homolog X-linked), *Sclerostin*, *Col1a1* (type I collagen), and *Runx2* (Runt-related transcription factor 2), which were all decreased in the melanoma metastasis group (Fig. 1c), supporting the reduction in trabecular and cortical bone volume that occurs as a result of melanoma bone metastasis. (Figs. S1E and F).

To delineate the molecular mechanisms underlying melanoma metastasis-induced osteocyte death, we performed bulk RNA sequencing of isolated mineralized bones to identify differentially expressed genes in osteocytes between the control and melanoma groups (Fig. S2A). Principal component analysis (PCA) revealed a remarkable divergence between these groups, indicating a significant disparity in their characteristics (Fig. 1d). Furthermore, applying Kyoto Encyclopedia of Genes and Genomes (KEGG) pathway enrichment analysis to the RNA sequencing data revealed a change in the ferroptosis pathway compared to other cell death pathways such as apoptosis and necroptosis (Fig. 1e). In addition, Gene Set Enrichment Analysis (GSEA) further confirmed the significant enrichment and activation of *‘response to iron ion’* in the melanoma metastatic bone (Fig. S2B). The heatmap of ferroptosis-related genes supported these findings (Fig. 1f), as the classical ferroptosis factor Gpx4 was downregulated.

In summary, our data suggests that melanoma metastasis in the bone triggers osteocyte death, likely leading to subsequent bone loss. Notably, among various cell death pathways, ferroptosis emerges as the primary mode of cell death in this context.

### Melanoma-derived conditioned medium (CM) induces osteocyte ferroptosis in vitro

We investigated how melanoma cells influence the death of osteocytes. First, we created melanoma-derived CM (Fig. S2C). Then, we used a cell-counting kit-8 assay to assess the viability of osteocytes (MLO-Y4) after being exposed to varying amounts of CM. The results showed a decrease in osteocyte viability that was proportional to the amount of melanoma-derived CM (Fig. S2D). In vitro, PI/Annexin V staining and TUNEL assays confirmed that melanoma-derived CM induced osteocyte death (Fig. S2E and F).

**Fig. 2:**
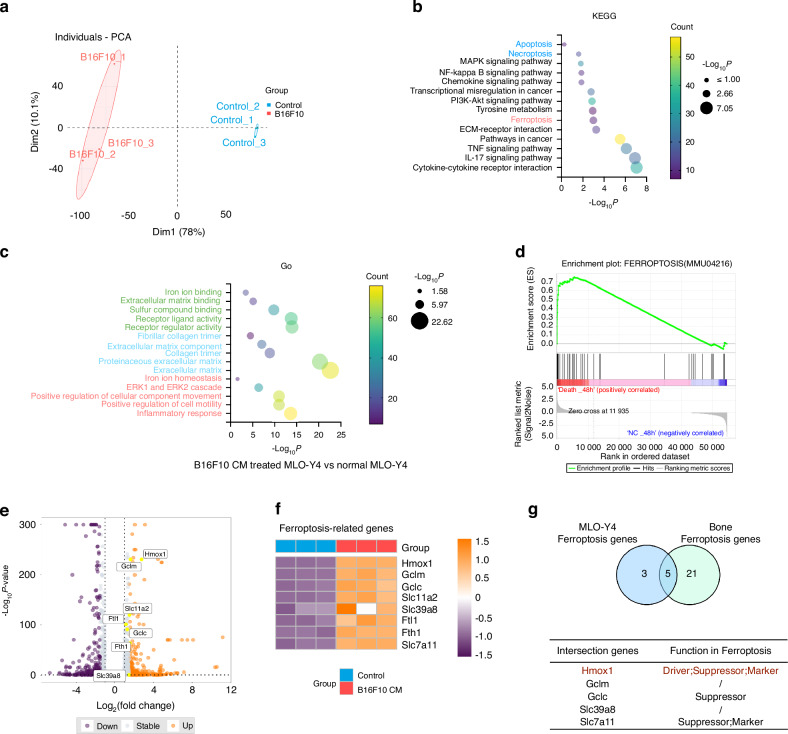
B16F10 Secretome-Induced Osteocyte Ferroptosis. **a** PCA plot showing sample patterns of individual samples in the control and B16F10-CM treated MLO-Y4 cells. **b** KEGG pathway analysis indicating “Ferroptosis” as the significantly altered pathway. **c** GO pathway analysis of B16F10-CM treated vs. untreated MLO-Y4 cells. **d** GSEA showing enrichment of the “Ferroptosis” pathway in B16F10-CM treated MLO-Y4 cells. **e**, **f** Volcano plot and heatmap of differentially expressed genes in ferroptosis pathways. **g**. Venn diagram of intersecting genes between bone-related and MLO-Y4 cell-related ferroptosis genes

RNA sequencing was used to assess the molecular mechanisms of osteocyte death induced by melanoma-derived CM (Fig. S2G). PCA exhibited the individual samples from two distinct groups (Fig. 2a), indicating a significant difference between these two groups. By using KEGG pathway enrichment analysis on the RNA sequencing data, we found a significant change in the ferroptosis pathway compared to other cell death pathways (Fig. 2b), which is consistent with our in vivo findings (Fig. 1e). Furthermore, the GO enrichment analysis highlighted pathways associated with iron, supporting the potential role of iron metabolism in this process (Fig. 2c). GSEA confirmed a positive correlation between osteocytes treated with melanoma-derived CM and the ‘Ferroptosis’ pathway (Fig. 2d). The genes implicated in the ‘Ferroptosis’ pathway in the melanoma-treated group are illustrated in the Volcano plot, while the Heatmap highlights genes that exhibit substantial alterations within this pathway (Fig. 2e and f). Furthermore, a Venn diagram was constructed comparing bone-related ferroptosis genes with MLO-Y4 cell-related ferroptosis genes, revealing five overlapping genes (Fig. 2g). Among these ferroptosis-related genes, Hmox1 showed the most significantly upregulated expression, both in vivo and in vitro, and emerged as a key player in ferroptosis regulation (Fig. 2g). Since Hmox1 has a pivotal role in heme oxidation and iron metabolism, crucial in ferroptosis,^24^ our findings suggest Hmox1 could play a critical role in melanoma metastasis-induced osteocyte ferroptosis.

### Importance of Hmox1 and Gpx4 in melanoma-induced osteocyte ferroptosis in vivo and in vitro

**Fig. 3:**
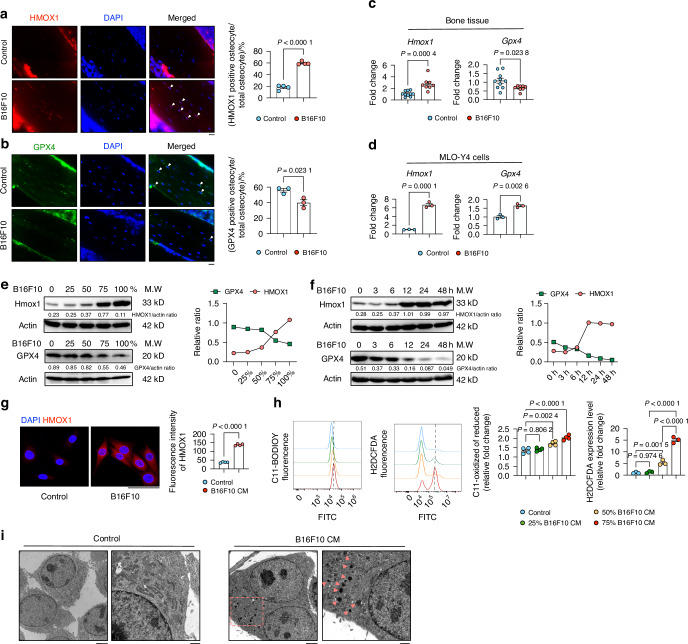
B16F10 Bone Metastasis Modulates HMOX1 and GPX4 Expression in Osteocytes. **a** HMOX1 staining and quantification in tibial bones from control and B16F10-injected mice (*n* = 4). Nuclei are stained with DAPI (blue). Scale bars: 20 μm. **b** GPX4 staining and quantification in tibial bones from control and B16F10-injected mice (*n* = 3). Nuclei are stained with DAPI (blue). Scale bars: 20 μm. **c**, **d** mRNA expression of *Hmox1* and *Gpx4* in tibial bones from control and B16F10-injected mice (n ≥ 6) (C), and in MLO-Y4 cells treated with or without 75% B16F10 CM for 48 h (*n* = 3) (D). **e** Western blot of HMOX1 and GPX4 in MLO-Y4 cells exposed to 0, 25%, 50%, 75%, and 100% B16F10 CM for 48 h. Actin was used as an internal control. **f** Western blot of HMOX1 and GPX4 in MLO-Y4 cells exposed to 75% B16F10 CM for 0-48 h. Actin was used as an internal control. **g** Confocal microscopy and quantification of HMOX1 in MLO-Y4 cells after 75% B16F10 CM treatment for 48 h. Scale bars: 50 μm. **h** FACS analysis of ferroptosis levels in MLO-Y4 cells after 48 h treatment with 0–75% B16F10 CM, using C11 BODIPY 581/591 and H2DCFDA. **i** Transmission electron microscopy (TEM) of MLO-Y4 cells treated with or without 75% B16F10 CM for 48 h. Scale bars: 2 500 nm (left), 1 000 nm (right). Statistical significance was determined by a 2-tailed Student’s t-test (A, B, C, D, G) or one-way ANOVA (H)

To further analyse melanoma-induced ferroptosis, Hmox1 and Gpx4 immunofluorescence (IF) was performed with bone tissue sections of tumor-bearing and control mice. A significant increase in Hmox1 expression within tibial osteocytes was observed in the melanoma group compared to controls (Fig. 3a). There was also a significant decrease in Gpx4-positive osteocytes in tumor metastatic bone (Fig. 3b). A similar trend was observed for *Hmox1* and *Gpx4* mRNA in bone tissue (Fig. 3c). Osteocytes treated with melanoma CM also showed increased *Hmox1* expression, but *Gpx4* expression was upregulated in melanoma CM-treated MLO-Y4 cells (Fig. 3d). Next, we performed a dose- and time-dependent exposure of osteocytes to melanoma-derived CM. A dose- and time-dependent increase in Hmox1 expression was coupled with a decrease in Gpx4 levels (Fig. 3e, f). Immunofluorescence assays for Hmox1 also confirmed the up-regulation of Hmox1 in melanoma CM-treated MLO-Y4 cells (Fig. 3g). To further quantify ferroptosis, two different ferroptosis probes, c11 BODIPY 581/591 and H2DCFDA, were used. These probes effectively demonstrated that melanoma-derived CM promoted osteocyte ferroptosis in a dose-dependent manner (Fig. 3h). The increased ferroptosis in melanoma CM-treated MLO-Y4 cells was confirmed by transmission electron microscopy (TEM) (Fig. 3i). Notably, there was a greater accumulation of lipid droplets in the cytoplasm of melanoma CM-treated MLO-Y4 cells compared to the untreated group (Fig. 3i).

Taken together, these findings showed an upregulation of Hmox1, downregulation of Gpx4, and an induction of ferroptosis in osteocytes by melanoma cells.

### Hmox1 inhibitor, Znpp, but not the classical ferroptosis inhibitor Fer-1, can rescue melanoma-induced osteocyte ferroptosis

**Fig. 4:**
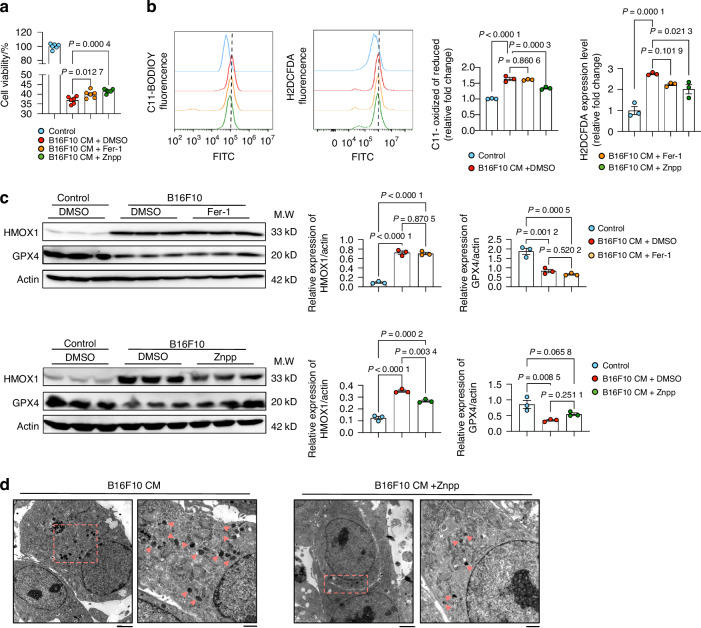
HMOX1 Inhibitor Znpp More Effectively Rescues B16F10-Induced MLO-Y4 Ferroptosis Than Fer-1. **a** MLO-Y4 cell viability after 48 h treatment with 75% B16F10 CM, with or without 24 h pretreatment with 10 μmol/L Fer-1, Znpp, or DMSO (control). **b** FACS analysis using C11 BODIPY 581/591 and H2DCFDA to quantify ferroptosis in MLO-Y4 cells treated as in (A). **c** Western blot of MLO-Y4 cells treated as in (A). Actin was used as an internal control. **d** Transmission electron microscopy (TEM) of MLO-Y4 cells after 48 h treatment with 75% B16F10 CM, with or without 24 h pretreatment with 10 μmol/L Znpp, or DMSO (control). Scale bars: 2 500 nm (left), 1 000 nm (right). Statistical significance was determined by one-way ANOVA

To verify the importance of Hmox1 in melanoma-derived CM-induced osteocyte ferroptosis, we used the ferroptosis inhibitor Fer-1 and the Hmox1 inhibitor Znpp. Osteocyte viability, assessed by CCK-8 assay, showed that both Fer-1 and Znpp treatments could partially protect against the osteocyte death induced by melanoma-derived CM (Fig. 4a). However, Znpp had a more potent effect than Fer-1 (Fig. 4a). Using the ferroptosis probe assay, we confirmed that Znpp treatment could partially alleviate melanoma-derived CM-induced osteocyte ferroptosis, better than Fer-1 (Fig. 4b). Znpp treatment significantly reduced the upregulation of Hmox1, induced by melanoma-derived CM in comparison to B16F10 + DMSO. However, no statistically significant differences in Gpx4 expression were evident between the Znpp and B16F10 + DMSO groups. (Fig. 4c). In contrast, Fer-1 treatment did not show significant differences in either Hmox1 or Gpx4 expression when compared to their respective controls (Fig. 4c). We thus conducted transmission electron microscopy (TEM) analysis and observed a greater accumulation of lipid droplets in the cytoplasm of MLO-Y4 cells treated with melanoma (Fig. 4d). Znpp treatment, however, significantly decreased the formation of these lipid droplets (Fig. 4d). The different results of Znpp and Fer-1 treatments suggest that the non-canonical ferroptosis pathway may be involved in melanoma-induced ferroptosis of osteocytes.

### Excessive autophagy induced autophagy-dependent ferroptosis

**Fig. 5:**
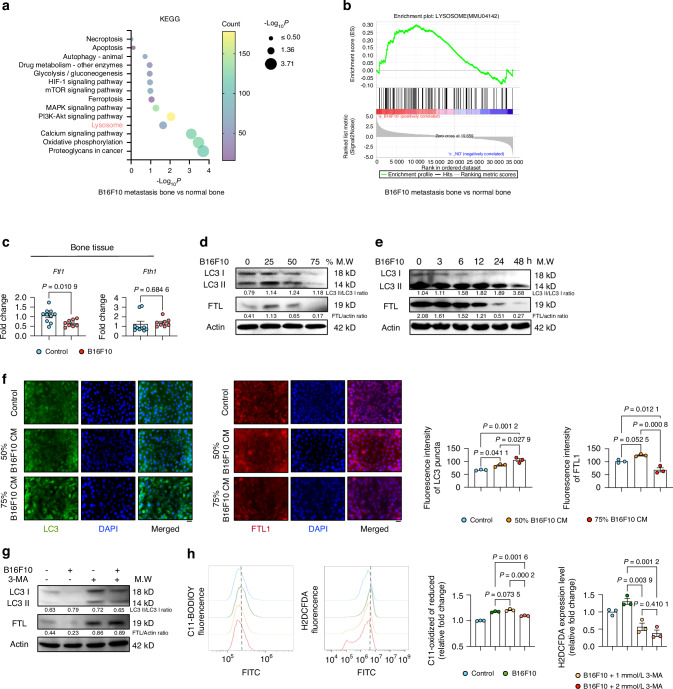
Autophagy in B16F10-Induced MLO-Y4 Ferroptosis Depends on HMOX1 Expression. **a** KEGG pathway analysis from bulk RNA-seq of long bone tissue (without bone marrow) from control or B16F10-injected mice, highlighting significant alteration in Lysosome. **b** GSEA showing enrichment of the “Lysosome” pathway in B16F10 group. **c** mRNA expression of Ftl1 and Fth1 in long bone tissue from control and B16F10-injected mice. **d** Western blot analysis of LC3 I, LC3 II, and FTL1 in MLO-Y4 cells treated with 0, 25%, 50%, and 75% B16F10 CM for 48 h. Actin was used as an internal control. **e** Western blot analysis of LC3 I, LC3 II, and FTL1 in MLO-Y4 cells treated with 75% B16F10 CM for 0 to 48 h. Actin was used as an internal control. **f** LC3 and FTL1 staining, with quantification of fluorescence intensity, in MLO-Y4 cells treated with or without 50% or 75% B16F10 CM for 48 h. Scale bars: 20 μm. **g** Western blot analysis of LC3 I, LC3 II, and FTL in MLO-Y4 cells treated with or without 75% B16F10 CM, and with or without 2 mmol/L 3-MA for 48 h. **h** FACS analysis using C11 BODIPY 581/591 and H2DCFDA to quantify ferroptosis in MLO-Y4 cells treated as in (**g**). Statistical significance was determined by a 2-tailed Student’s t-test (**c**) or one-way ANOVA (**f**, **h**)

The relationship between autophagy and ferroptosis is complex. Excessive autophagy-driven degradation of ferritin can facilitate ferroptosis.^25–27^ Interestingly, bulk RNA sequencing from bone tissues (Fig. S2A) showed a significant enrichment of the ‘Lysosome’ pathway associated with melanoma metastasis (Fig. 5a). Moreover, GSEA showed a positive correlation between melanoma metastasis and the ‘Lysosome’ phenotype (Fig. 5b). The initial findings highlight the significant impact of autophagy, specifically through lysosomal pathways,^28^ on melanoma metastasis in bone tissues. Analysis of bone tissues using qPCR showed that melanoma metastasis caused degradation of ferritin light chain 1 (*Ftl1*), while ferritin heavy chain 1 (*Fth1*) was not affected (Fig. 5c). In an in vitro experiment, MLO-Y4 cells were exposed to melanoma-derived CM in a dose- and time-dependent manner. The results showed a gradual increase in the LC3II/LC3 I ratio, despite the overall decrease in LC3 levels (Fig. 5d and e). It suggests that LC3-I is converted to LC3-II at an accelerated rate, indicating an increased turnover of LC3-II after autophagosome formation. Although the conversion rate appears faster, the net levels of both LC3 forms decrease, indicating an elevated autophagic flux.^29^ In particular, an initial upregulation of Ftl1 was observed at lower CM concentrations, followed by degradation at higher concentrations and prolonged CM exposure (Fig. 5d and e). IF intensity of LC3 correlated with the concentration of melanoma-derived CM, while Ftl1 intensity showed an initial increase with exposure to 50% CM and a decrease in Ftl1 intensity with 75% CM (Fig. 5f). Because of the correlation between excessive autophagy-induced ferritin degradation and the promotion of ferroptosis, we hypothesized that 3-MA, a classical autophagy inhibitor, could attenuate melanoma-induced ferroptosis. 3-MA treatments resulted in a reduction of excessive autophagic flux, as indicated by the decreased LC3II/LC3 I ratio (Fig. 5g). Consequently, the degradation of Ftl1 was rescued by 3-MA treatments (Fig. 5g). In accordance, a significant reduction in melanoma-induced ferroptosis was observed upon 3-MA treatments (Fig. 5h). Collectively, these data suggest that osteocyte ferroptosis induced by melanoma-derived CM functions as a non-canonical ferroptosis dependent on autophagy mechanisms.

### Hmox1 is essential in autophagy-dependent ferroptosis of osteocytes

**Fig. 6:**
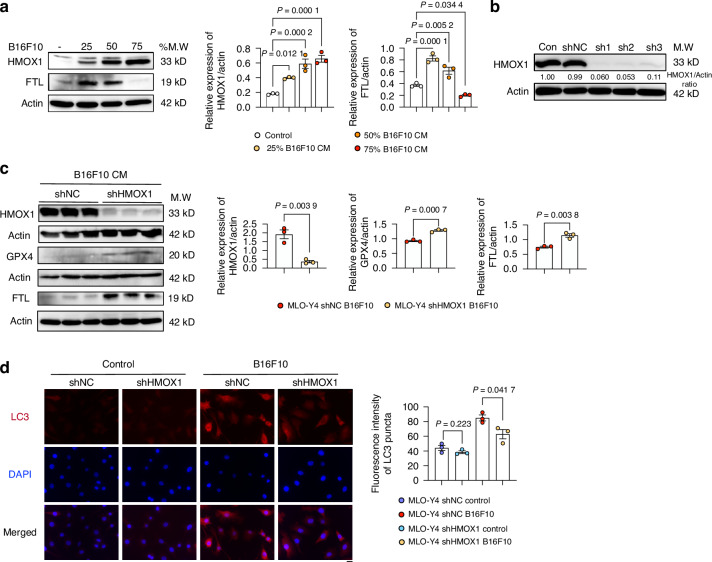
Hmox1 Knockdown Rescues B16F10-Induced MLO-Y4 Autophagy-Dependent Ferroptosis. **a** Western blot analysis of HMOX1 and FTL in MLO-Y4 cells treated with 0%, 25%, 50%, and 75% B16F10 CM for 48 h. Actin was used as an internal control. **b** Western blot of HMOX1 in MLO-Y4 cells transfected with scrambled shRNA (shNC) or three different shRNAs targeting HMOX1. Actin was used as an internal control. **c** Western blot HMOX1, GPX4, and FTL in MLO-Y4 cells transfected with shNC or shRNA-HMOX1, and exposed to 75% B16F10 CM for 48 h. Actin was used as an internal control. **d** Immunofluorescence of LC3 staining and quantification of LC3 fluorescence intensity in MLO-Y4 cells transfected with shNC or shRNA-HMOX1, with or without 48 h exposure to 75% B16F10 CM. Scale bars: 20 μm. Statistical significance was determined by a 2-tailed Student’s t-test (**c**, **d**) or one-way ANOVA (A)

We next investigated the role of Hmox1 in autophagy-dependent osteocyte ferroptosis. Western blot analysis revealed an accumulation of Hmox1 in osteocytes after an increased percentage of CM stimulation (Fig. 6a). An initial increase followed by a subsequent decrease in Ftl1 levels was also observed in CM-treated MLO-Y4 (Fig. 6a). Next, we generated Hmox1 knockdown in osteocytes using shRNA (Fig. 6b) and exposed the cells to melanoma-derived CM. The knockdown of Hmox1 reversed the effects of melanoma-derived conditioned medium (CM), including the reduction in Gpx4 levels and the degradation of Ftl1 (Fig. 6c). Furthermore, Hmox1 knockdown reduced the excessive autophagy induced by melanoma-derived CM (Fig. 6d). Taken together, our results suggest that Hmox1 is the primary factor controlling autophagy-dependent osteocyte ferroptosis.

### Hmox1 inhibitor, Znpp, rescued melanoma-induced autophagy-dependent ferroptosis in vivo

**Fig. 7:**
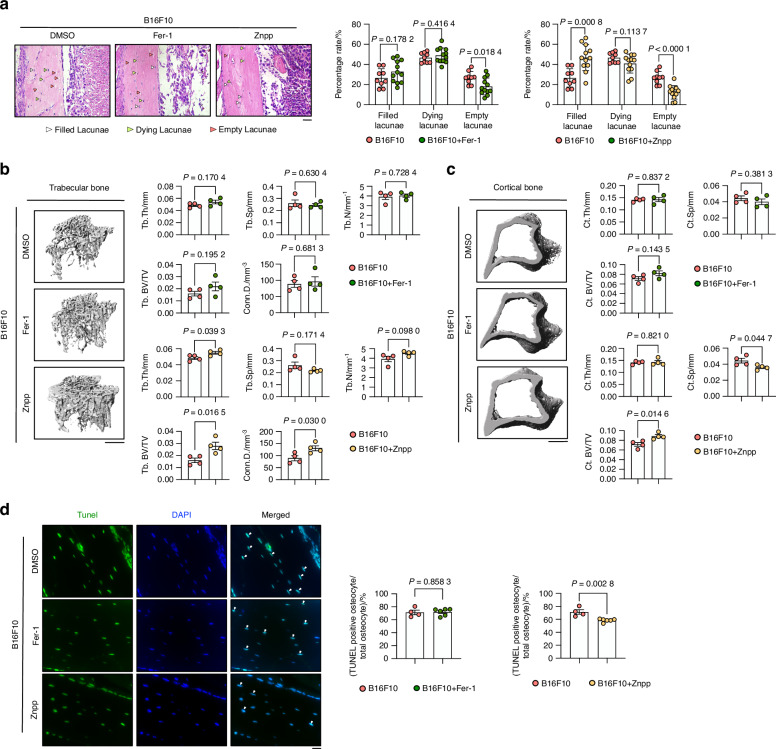
Znpp Rescues B16F10-Induced Autophagy-Dependent Ferroptosis In Vivo. **a** H&E staining of cortical bones from mice injected with B16F10 and treated with DMSO, 1 mg/kg Fer-1, or 10 mg/kg Znpp (i.p.). Quantification of filled, dying, and empty lacunae (*n* ≥ 8 per group). White arrows: normal osteocytes; green arrows: dying osteocytes; red arrows: dead osteocytes. Scale bars: 20 μm. **b** µCT analysis of trabecular bone from mice described in (A), including bone volume per total volume (Tb.BV/TV), separation (Tb.Sp), number (Tb.N), thickness (Tb.Th), and connective density (Conn.D) in tibial bones (*n* = 4 per group). **c** µCT analysis of cortical bone mice described in (A), including bone volume per total volume (Ct.BV/TV), separation (Ct.Sp), and thickness (Ct.Th) in tibial bones (*n* = 4 per group). **d** TUNEL staining and quantification of TUNEL-positive cells in cortical bones (*n* ≥ 4 per group) mice described in (A). White arrows indicate TUNEL-positive cells. Statistical significance was determined by a 2-tailed Student’s t-test

**Fig. 8:**
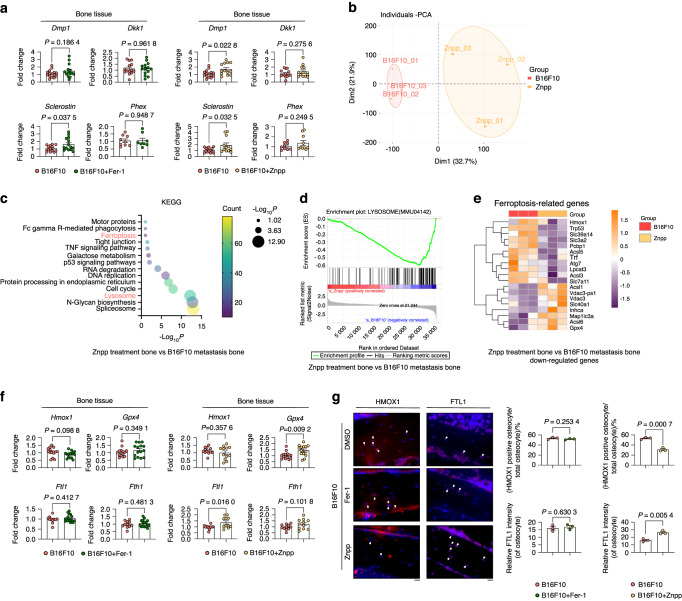
Znpp Rescues B16F10-Induced Autophagy-Dependent Ferroptosis In Vivo. **a** mRNA expression of Dmp1, Dkk1, Phex, and Sclerostin in long bone tissue (without bone marrow) from mice injected with B16F10 and treated with DMSO, 1 mg/kg Fer-1, or 10 mg/kg Znpp (i.p.) (*n* ≥ 8 per group). **b**–**e** Bulk RNA-seq analysis of long bone tissue (without bone marrow) from mice injected with B16F10, treated or not with 10 mg/kg Znpp (*n* = 3 per group). **b** PCA showing patterns of individual samples in the B16F10 and Znpp-treated groups. **c** KEGG pathway analysis of downregulated DEGs, highlighting significantly altered pathways. **d** GSEA of downregulated DEGs showing enrichment of the lysosome pathway in the Znpp treatment group. **e** Heatmap displaying ferroptosis pathway-associated DEGs between the groups. **f** mRNA expression of Hmox1, Gpx4, Ftl1, and Fth1 in long bone tissue (without bone marrow) from mice described in (A) (*n* ≥ 10 per group). **g** HMOX1 IF staining and FTL1 positive cell quantification in the proximal tibia from mice described in (A) (*n* = 3 per group). Scale bars: 20 μm. Statistical significance was determined by a 2-tailed Student’s t-test

To translate our findings in vivo, we injected the ferroptosis inhibitors Fer1 or Znpp during the bone metastasis model (Fig. S3A). Body weight assessment revealed a rescue of the weight loss induced by melanoma injection after Fer1 or Znpp treatments (Fig. S3B). H&E staining to assess bone tumor growth showed that neither Fer-1 nor Znpp had a significant effect on melanoma bone metastasis (Fig. S3C). We then performed histochemical analysis to assess the rate of osteocyte death. Figure 7a shows a lower percentage of empty lacunae (dead osteocytes) and a higher percentage of filled lacunae (normal osteocytes) in mice treated with Znpp, but no difference after Fer1 treatment. While Znpp treatment successfully counteracted the reduction in trabecular BV/TV, Tb. Th and Conn.D in the melanoma group, Fer-1 treatment did not produce similar effects (Fig. 7b). Furthermore, the effects of the two inhibitors on cortical bone (Fig. 7c) were analysed. Only Znpp treatment resulted in a reduction of the cortical BV/TV and a decrease in melanoma-induced Ct.sp up-regulation. In contrast, Fer-1 showed no effect (Fig. 7c). Consistent with this, a decreased number of TUNEL-positive cells was observed in the Znpp treatment group compared to the melanoma group, whereas the Fer-1 treatment had no such effect on osteocyte cell death (Fig. 7d). qPCR analysis of bone tissue provided additional confirmation of the beneficial effect of Znpp treatment on melanoma-induced osteocyte loss, as shown in Fig. 8a. We also examined several cell death related markers, including *Bax* (BCL2-associated X protein), caspase 3 (*Casp3*) and caspase 8 (*Casp8*), which confirmed that only Znpp treatment could mitigate melanoma bone metastasis-induced osteocyte death (Fig. S3D). Bone tissue bulk RNA sequencing after Znpp treated mice was compared to RNA seq from untreated metastatic mice. PCA showed individual samples from the two distinct groups (Fig. 8b). Subsequent KEGG pathway enrichment analysis, focusing on down-regulated differentially expressed genes (DEGs), highlighted a significant alteration in the ‘Lysosome’ pathway (Fig. 8c). The heatmap representation (Fig. S3E) displayed the down-regulated DEGs related to lysosome function. GSEA for the DEGs further corroborated the negative association of the ‘Lysosome’ pathway in the Znpp treatment group (Fig. 8d). The heatmap representation of the ferroptosis-related gene expression corroborated the finding that Znpp treatment decreased ferroptosis genes when compared to untreated samples (Fig. 8e). Additionally, qPCR analyses of bone tissues showed a decreased expression level of *Gpx4* and *Ftl1* after Znpp treatment, while no difference after Fer1 treatment (Fig. 8f). Furthermore, IF was performed for Hmox1 and Ftl1, which demonstrated that Znpp treatment led to a reduction in the expression of Hmox1 and an alleviation of the degradation of Ftl1 (Fig. 8g).

To ascertain whether the observed effects on bone mass were attributable to osteoclast activity, we analysed their differentiation and activity. As illustrated in Fig. S4A, osteoclast culture demonstrated that melanoma-conditioned medium reduced osteoclast differentiation. The results were further corroborated by qPCR analysis in Fig. S4B, which demonstrated that a low concentration of melanoma-derived CM markedly reduced the expression of core markers associated with osteoclasts. Our previous studies demonstrated a notable reduction in osteoclast numbers in the melanoma bone metastasis model in comparison to the control group (i.e., the group without tumor formation).^30^ As shown in Fig. S4C, neither Fer-1 nor Znpp treatment was able to reverse the reduction in osteoclast numbers resulting from B16F10 bone metastasis. These findings indicate that the alterations in bone mass and osteocyte survival observed with Znpp treatment are not contingent on osteoclast activity.

These data showed that Hmox1 inhibition alleviated melanoma-induced autophagy-dependent osteocyte ferroptosis.

### Hif1α signaling pathway is up-stream of Hmox1 in melanoma-induced autophagy-dependent ferroptosis

**Fig. 9:**
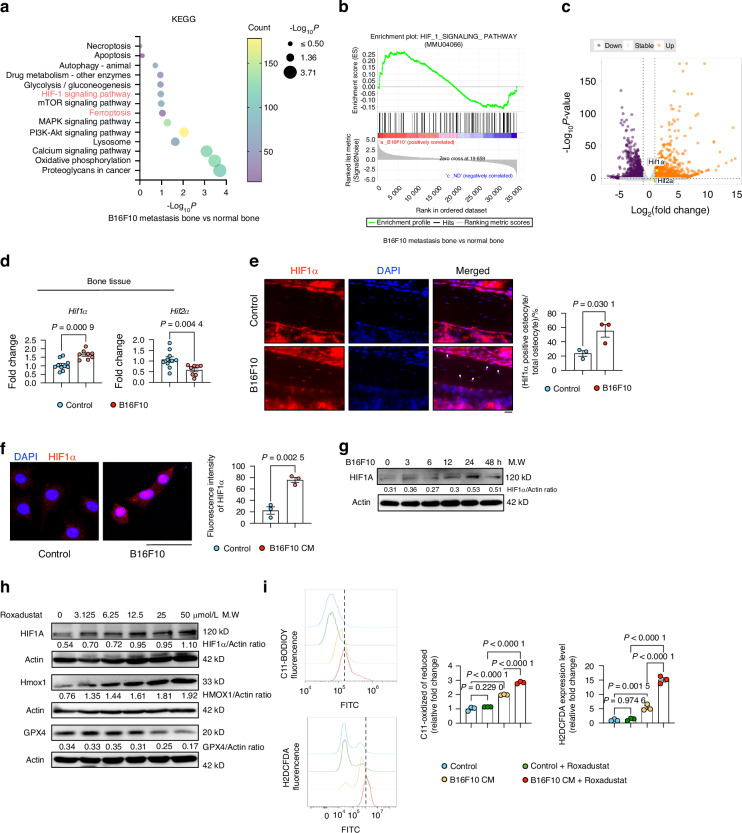
Upregulation of HIF1α Signaling in Osteocytes After B16F10 Exposure. **a** KEGG pathway analysis from bulk RNA-seq of long bone tissue (without bone marrow) from control or B16F10-injected mice, highlighting significant alteration in HIF1α signaling pathway. **b** GSEA showing enrichment of the ‘HIF1α signaling pathway’ in the B16F10 group. **c** Volcano plot analysis displaying differentially expressed genes. **d** mRNA expression of Hif1α and Hif2α in long bone tissue from control or B16F10-injected mice (*n* ≥ 6). **e** HIF1α immunofluorescence staining and quantification in proximal tibia from control and B16F10-injected mice. Nuclei are visualized by DAPI (blue). White arrows indicate HIF1α-positive cells (*n* = 3). Scale bars: 20 μm. **f** Confocal microscopy of HIF1α in MLO-Y4 cells treated with or without 75% B16F10-derived CM for 48 h. Scale bars: 50 μm. **g** Western blot analysis of HIF1α in MLO-Y4 cells exposed to 75% B16F10-derived CM for 0-48 h. Actin was used as an internal control. **h** Western blot analysis of HIF1α, HMOX1, and GPX4 in MLO-Y4 cells treated with varying doses of Roxadustat (HIF1α stabilizer) for 48 h. Actin was used as an internal control. **i** FACS analysis of C11 BODIPY 581/591 and H2DCFDA to assess ferroptosis levels in MLO-Y4 cells with or without 75% B16F10-derived CM, treated with or without 50 μmol/L Roxadustat treatment for 48 h. Statistical significance was determined by a 2-tailed Student’s t-test for single comparisons (**d**, **e**, **f**) and one-way ANOVA for multiple comparisons (I)

To delineate the upstream pathway of Hmox1, RNA sequencing data comparing melanoma metastatic bone to normal bone was analysed (Fig. S2A). The analysis revealed a strong enrichment of the ‘Hif1 pathway’ in bone metastasis (Fig. 9a). GSEA revealed a positive correlation between the Hif1 pathway and melanoma metastasis (Fig. 9b). Remarkably, the volcano plot highlighted a significant upregulation of Hif1α expression (Fig. 9c), which was validated by qPCR on bone tissue (Fig. 9d). Despite the lack of discernible variation in Hif2α expression among the melanoma metastasis cohort in the volcano plot, the qPCR analysis did reveal a reduction in the melanoma group (Fig. 9d). IF analysis on bone tissue showed increased Hif1α expression levels in the melanoma group (Fig. 9e). Confocal staining and western blot of Hif1α in MLO-Y4 stimulated with melanoma CM showed an up-regulation of Hif1α after treatment with melanoma-derived CM (Fig. 9f and g).

To investigate the effect of Hif1α in autophagy-dependent osteocyte ferroptosis, we used Roxadustat, a small molecule Hif stabilizer that inhibits prolyl hydroxylase (Phd).^31^ Western blot analysis showed that Roxadustat significantly increased Hif1α levels in MLO-Y4 (Fig. 9h). Regarding Hif2α, a low concentration of Roxadustat promoted its expression, whereas the high concentration employed in the subsequent experiments returned it to baseline levels (Fig. S5A), thereby excluding the influence of Hif2α. Roxadustat significantly increased Hmox1 levels while decreasing Gpx4 expression (Fig. 9h). Roxadustat exacerbated melanoma-derived CM-induced osteocyte death (Fig. S5B) and promoted further ferroptosis induced by melanoma-derived CM (Fig. 9i). PI/Annexin V staining revealed that Roxadustat not only induced melanoma-induced osteocyte ferroptosis but also induced apoptosis (Fig. S5C). The findings indicate that Roxadustat induces osteocyte ferroptosis and apoptosis in the presence of melanoma-derived CM. Similar observations were made using an overexpressing Hif1α model (Fig. S6A), with Hif1α overexpression further enhancing melanoma-derived CM-induced ferroptosis (Fig. S6B) and apoptosis (Fig. S6C). Further, knockdown of Hif1α in MLO-Y4 cells (Fig. S6A) reversed melanoma-derived CM-induced ferroptosis (Fig. S6D) and apoptosis (Fig. S6E). These findings suggests that Hif1α signaling pathway is involved in melanoma-induced autophagy-dependent ferroptosis in vitro.

### Roxadustat promoted melanoma-induced autophagy-dependent osteocyte ferroptosis in vivo

**Fig. 10:**
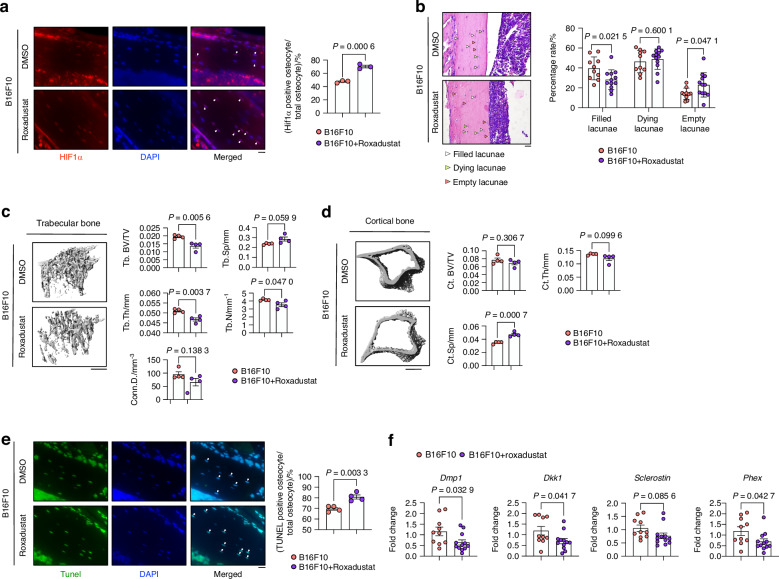
Roxadustat Enhances B16F10-Induced Ferroptosis in Osteocytes. **a** HIF1α immunofluorescence staining and quantification of HIF1α-positive cells in proximal tibia from B16F10-injected mice treated with DMSO or 10 mg/kg Roxadustat (i.p.) daily. Scale bars: 20 μm. **b** H&E staining of cortical bone from mice described in (A). Quantification of filled, dying, and empty lacunae (*n* ≥ 8 per group). White arrows: normal osteocytes; green arrows: dying osteocytes; red arrows: dead osteocytes. Scale bars: 20 μm. **c** µCT analysis of trabecular bone from mice described in (A), showing trabecular bone volume (Tb.BV/TV), separation (Tb.Sp), number (Tb.N), thickness (Tb.Th), and connectivity density. **d** µCT analysis of cortical bone from mice described in (A), showing cortical bone volume (Ct.BV/TV), separation (Ct.Sp), and thickness (Ct.Th). **e** TUNEL staining and quantification of TUNEL-positive cells in cortical bone from mice described in (A). Scale bars: 20 μm. **f** mRNA expression of Dmp1, Dkk1, Sclerostin, and Phex in long bone tissue from mice described in (A). Statistical significance was determined by a 2-tailed Student’s t-test

Next, our in vivo assessment was conducted with the aim of evaluating the efficacy of Roxadustat (Fig. S7A). Following intracardiac injection of melanoma cells, mice were either untreated or treated with 10 mg/kg Roxadustat daily for 14 days. Body weight measurements show a weight loss in the Roxadustat-treated group compared to the melanoma group (Fig. S7B). Immunofluorescence of Hif1α confirmed its upregulation in the Roxadustat group compared to the melanoma group (Fig. 10a). Furthermore, IF of Hif2α revealed that Roxadustat did not alter Hif2α expression in osteocytes, and that melanoma metastasis also did not change Hif2α expression (Fig. S7C). Melanoma bone metastasis rates indicated no impact of Roxadustat treatment on the tumor volume in the tibia or femur (Fig. S7D). Furthermore, additional evaluation of tumor volume in the lung demonstrated no notable discrepancy between the Roxadustat treatment group and the control group. (Fig. S7D). Moreover, mice treated with Roxadustat during melanoma metastasis demonstrated a higher percentage of empty lacunae (an indicator of osteocyte death) and a lower percentage of filled lacunae (an indicator of normal osteocyte function) (Fig. 10b). However, Roxadustat did not influence osteocyte survival rates under normal conditions (Fig. S7E). Roxadustat treatment exacerbated bone loss as shown with reduction in Tb.Th and Tb.N compared to the melanoma group (Fig. 10c). Further analysis of the impact of Roxadustat on cortical bone revealed that Roxadustat markedly enhanced melanoma-induced Ct.sp up-regulation (Fig. 10d). Next, an increased number of TUNEL-positive cells was quantified in the Roxadustat treatment group compared to the melanoma group (Fig. 10e). qPCR analysis of bone tissues revealed that treatment with Roxadustat under standard conditions (Control+Roxadustat) did not result in any discernible impact on the osteocyte markers, whereas injection of B16F10 reduced their levels (Fig. S7F). In the context of melanoma metastasis, Roxadustat treatment decreased these markers (Fig. 10f) and increased cell death markers (Fig. S7G).

**Fig. 11:**
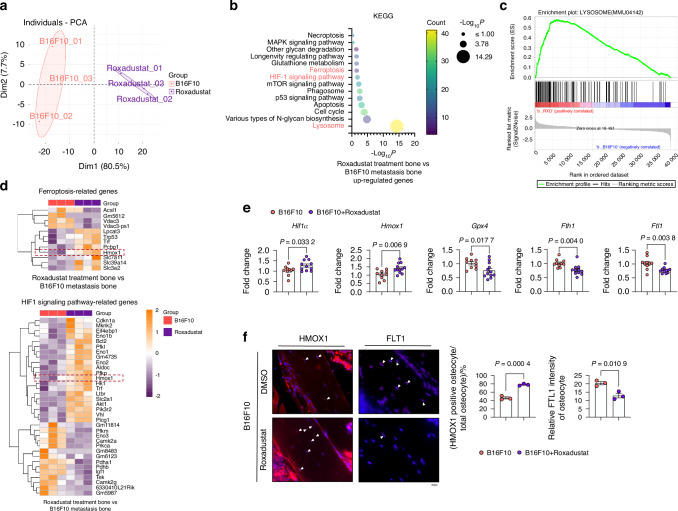
Roxadustat Enhances B16F10-Induced Autophagy-Dependent Ferroptosis in Osteocytes. **a**–**d** Bulk RNA-seq analysis of long bone tissue (without bone marrow) from mice injected with B16F10 and treated with DMSO or 10 mg/kg Roxadustat daily (i.p.) (*n* = 3 per group). **a** PCA plot showing sample patterns of individual samples in the B16F10 and Roxadustat-treated groups. **b** KEGG pathway analysis of up-regulated DEGs, highlighting significantly altered pathways. **c** GSEA of up-regulated DEGs showing enrichment in the lysosome pathway in Roxadustat-treated group. **d** Heatmap of DEGs associated with Ferroptosis and HIF1 signaling pathways. **e** mRNA expression of Hif1α, Hmox1, Gpx4, Ftl1, and Fth1 in long bone tissue from mice described in (A) (*n* ≥ 10 per group). **f** HMOX1 immunofluorescence staining and quantification of FTL1-positive cells in the proximal tibia from the mice described in (A). Scale bars: 20 μm. Statistical significance was determined by a 2-tailed Student’s t-test

To investigate the molecular mechanisms of Roxadustat on metastasis-induced osteocyte death, RNA sequencing was done in isolated bone tissue. PCA indicated a significantly distant tendency between Roxadustat and untreated group (Fig. 11a). KEGG pathway enrichment analysis, focusing on up-regulated DEGs, highlighted a significant alteration in the ‘Lysosome’ pathway, as well as ‘Hif1 signaling pathway’ and ‘Ferroptosis’ (Fig. 11b). Additionally, GSEA confirmed the positive association of the ‘Lysosome’ pathway in the Roxadustat treatment (Fig. 11c). The heatmap representation illustrated up-regulated DEGs linked to lysosome function (Fig. S8A), ferroptosis-related genes and Hif1 signaling pathway (Fig. 11d). Hmox1 was identified expressed in both signaling pathways (Fig. 11d). The expression levels of *Hif1a* and *Hmox1* were increased in the Roxadustat treated group, while *Gpx4, Fth1* and *Ftl1* expression was reduced (Fig. 11e). IF analyses for Hmox1 and Ftl1 revealed that Roxadustat treatment promoted the expression of Hmox1 and caused the degradation of Ftl1 compared to the melanoma group (Fig. 11f).

In addition, Fig. S8B demonstrated that Roxadustat treatment was unable to reverse the reduction in osteoclast numbers resulting from B16F10 bone metastasis. These findings indicate that the alterations in bone mass and osteocyte survival observed with Roxadustat treatment are not contingent on osteoclast activity.

Our data demonstrates that Roxadustat specifically exacerbates melanoma-induced autophagy-dependent osteocyte ferroptosis, consistent with our in vitro findings.

### Identified Hif1α as the up-stream transcription factor of Hmox1

**Fig. 12:**
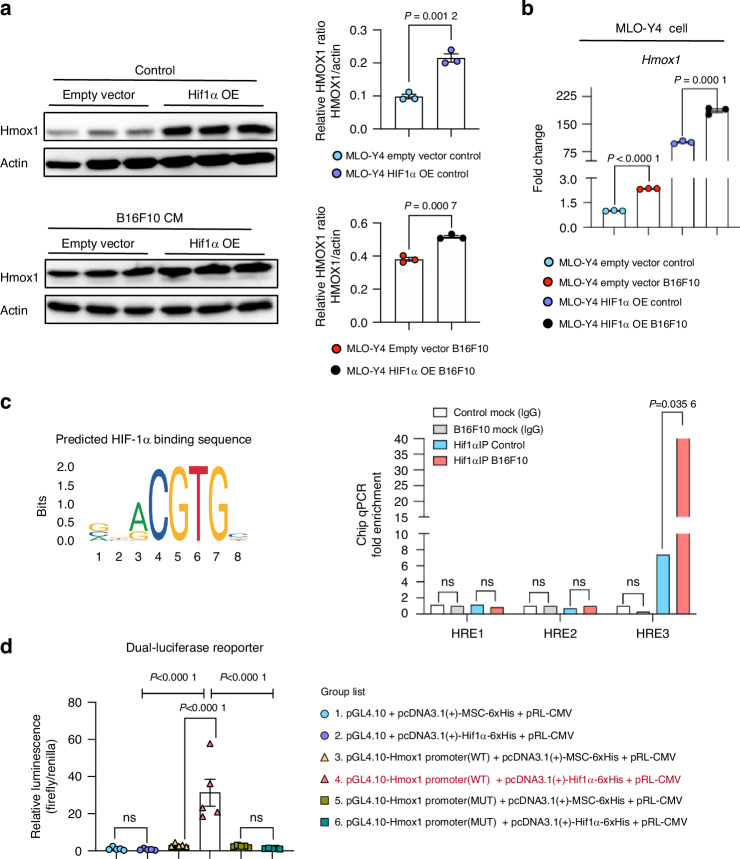
HIF1α Transcriptionally Controls Hmox1 Expression. **a** Western blot of HMOX1 in MLO-Y4 cells transfected with either an empty vector or a HIF1α overexpression plasmid, under normal conditions (upper) or with B16F10 CM treatment (lower). Actin served as an internal control. **b** mRNA expression of Hmox1 in MLO-Y4 cells transfected with empty vector or HIF1α overexpression plasmid, with or without 75% B16F10-derived CM for 48 h. **c** CHIP qPCR assays showing HIF1α binding to the Hmox1 promoter in MLO-Y4 cells. A transcription factor HIF1α binding sequence from the JASPAR database is shown (left). **d** Luciferase reporter assay in HEK 293 T cells co-transfected with the indicated plasmids to assess HIF1α’s effect on the Hmox1 promoter activity. Groups as mentioned in figure. Statistical significance was determined by a 2-tailed Student’s t-test for single comparisons (A, B, C) and one-way ANOVA for multiple comparisons (D)

To further elucidate the connection between Hif1α and Hmox1, we subjected MLO-Y4 cells to Hif1α overexpression or an empty vector, followed by exposure to melanoma-derived CM or no treatment. Hif1α overexpression significantly increased Hmox1 expression in untreated cells, and after treatment with melanoma-derived CM (Fig. 12a and b). Next, we delineate whether *Hmox1* gene expression is transcriptionally regulated by Hif1α. Bioinformatics promoter analysis utilizing the JASPAR database identified the predicted Hif1α binding sequence on *Hmox1* promoter (Fig. 12c). Chromatin immunoprecipitation (ChIP) assay demonstrated that Hif1α indeed bound to the HREs within 5000 bp upstream of the promoter region (Fig. 12c), suggesting a direct transcriptional regulation of *Hmox1* by Hif1α through specific binding to the region 3 in its promoter. Subsequently, a dual-luciferase reporter assay was conducted to assess the impact of Hif1α on *Hmox1* promoter activity. As illustrated in Fig. 12d, Hif1α overexpression does not influence the luminescence of the empty pGL4.10 vector (group 1 vs. group 2). However, the combination of Hif1α overexpression on the wild-type *Hmox1* promoter (group 4) resulted in a significant increase in luminescence compared to the wild-type *Hmox1* promoter without Hif1α overexpression (group 3), indicating that Hif1α significantly activates the *Hmox1* promoter. To further verify the activation effect of Hif1α on the *Hmox1* promoter, we used the mutant promoter plasmid (pGL4.10-*Hmox1* promoter (MUT)). The data indicated that Hif1α overexpression alone does not affect the luminescence of the mutation groups (group 5 vs. group 6). However, the mutation in the *Hmox1* promoter abrogates Hif1α-induced activation (group 4 vs. group 6). In conclusion, these findings suggest a direct transcriptional regulation of Hmox1 by Hif1α in MLO-Y4 cells.

## Discussion

Our results show that ferroptosis is the key driver of osteocyte death in melanoma bone metastasis. We have identified Hmox1 as a key regulatory element in this process, directly triggering ferroptosis and affecting osteocyte survival. We also reveal an unconventional pathway involving excessive autophagy-driven ferritin degradation, highlighting the intricate interplay between autophagy and ferroptosis in the context of melanoma-related osteocyte mortality. Furthermore, the Hif1α pathway is established as an upstream modulator and represents a potential target to modulate Hmox1 expression and influence autophagy-dependent ferroptosis.

Previous studies have shown that melanoma cells in the bone microenvironment can disrupt the osteocyte network.^7,15^ As previously observed in other bone metastasis models, we observed that tumor cells trigger osteolytic bone destruction by increasing osteocyte death.^32,33^ Our investigation has provided compelling evidence of osteocyte death induced by melanoma bone metastasis, validated by extensive in vivo and in vitro experiments. RNA sequencing of isolated bone and pathway enrichment analyses delineate the molecular landscape and reveal a clear shift towards ferroptosis as the primary mechanism governing melanoma metastasis-induced osteocyte death. The importance of the ferroptosis pathway in osteocytes has not yet been elucidated in bone melanoma metastasis, but was previously shown in mice with ovariectomy (OVX)-induced osteoporosis, mainly due to iron accumulation in bone tissue.^34^

Extending our in vivo findings, our in vitro experiments using melanoma-derived conditioned medium (CM) provided additional evidence for the induction of osteocyte ferroptosis. In particular, melanoma-induced osteocyte ferroptosis is specifically dependent on Hmox1, a known factor in heme oxidation and iron metabolism important for ferroptosis.^35–37^ By specifically targeting Hmox1 with the Znpp inhibitor or shRNA, our results clearly confirm its critical regulatory role both in vivo and in vitro and firmly link it to the regulation of ferroptosis. A direct implication of Hmox1 expression in bone remodeling and communication between bone and prostate tumor cells has previously been suggested,^38^ without determining its importance in osteocytes. Our investigation revealed intriguing complexities in the rescue of melanoma-induced ferroptosis. While classical ferroptosis inhibitors such as Fer-1 showed efficacy, the superior potential of the Hmox1 inhibitor Znpp challenged the canonical understanding of ferroptosis in this context. These unexpected findings, together with revelations regarding the role of autophagy in modulating ferroptosis, revealed a non-canonical pathway governing melanoma-induced osteocyte death.

In physiological conditions, lysosomes within osteocytes play a pivotal role in maintaining cellular homeostasis, bone remodeling processes, managing calcium dynamics, responding to mechanical stimuli, and integrating signaling pathways essential for bone metabolism.^39,40^ The lysosome is of particular importance for osteocyte function, as it plays a pivotal role in the osteocyte response to mechanical signals. For example, sclerostin is degraded by the lysosome following exposure to bone anabolic stimuli.^41^ In pathological conditions such as cancer and inflammation, there are notable alterations in the functionality of lysosomes in osteocytes. In such contexts, lysosomes assume a pivotal role in the final stages of autophagy, facilitating the degradation and recycling of cellular components.^42^ Additionally, lysosomes contribute significantly to the regulation of iron homeostasis by modulating the release of iron from ferritin, a critical process implicated in various disease states.^43^ In accordance with our findings, previous research has shown a link between ferroptosis and autophagy-dependent cell death processes,^26,44,45^ with excessive autophagy triggering ferritin degradation.^27,46,47^ The upregulation of autophagy-related genes and subsequent ferritin degradation highlights the dual role of autophagy in iron metabolism, first as a protective mechanism followed by its potential contribution to ferroptosis.

In addition, our investigation of upstream regulators of the autophagy/ferroptosis axis identified the Hif1α transcription factor as a critical contributor. Previous studies have shown that Roxadustat-induced stabilization of Hif1α suppresses chemoresistant glioblastoma growth by inducing ferroptosis.^48^ Increased Hif1α expression has been consistently associated with the promotion of ferroptosis in various diseases.^49,50^ Hif1α expression was reported as a key regulator involved in hypoxic apoptosis of MLO-Y4 osteocytes^51^ and it also plays a complex role in bone biology, exhibiting both protective and inhibitory actions under different conditions. For instance, stabilizing Hif1α isoforms causes high bone mass phenotypes during bone development.^52^ Conversely, in some pathological conditions, Hif1α activation enhances osteoclast-mediated bone resorption, resulting in bone loss.^53^ Hif1α plays also a dual role in senile osteoporosis.^54^ These findings underscore Hif1α‘s complex role in different disease contexts. In osteocytes, the function of Hif1α is still controversial. Hypoxic conditions can induce MLO-Y4 osteocyte apoptosis,^51^ whereas Roxadustat-induced upregulation of Hif1α in osteocyte cell lines significantly decreased the expression of osteocyte-related genes such as *Dmp1* and *Phex*.^55^ However, overexpression of Hif1α has also been shown to protect bone cells from dexamethasone-induced apoptosis.^56^ These findings underscore the dualistic nature of Hif1α, acting either as a promoter or inhibitor of bone formation depending on the specific context. In our study, we highlight the regulatory role of the Hif1α/Hmox1 axis in ferroptosis across various biological settings. Similar to our analyses, Hif1α was shown to be an upstream transcription factor for Hmox1, influencing ferroptosis processes in diverse cell types such as vascular smooth muscle cells^57^ and mouse testes exposed to Di(2-ethylhexyl) phthalate (DEHP).^58^ These studies underscore the versatility of the Hif1α/Hmox1 axis in modulating ferroptotic pathways under different physiological and pathological conditions.

In the context of melanoma-induced osteocyte ferroptosis, our findings suggest that the Hif1α/Hmox1 axis plays a crucial role. Specifically, we identify an unconventional pathway involving excessive autophagy-driven ferritin degradation as a mechanism contributing to osteocyte death in melanoma bone metastasis. This pathway, mediated by Hif1α and Hmox1, represents a novel aspect of ferroptosis regulation in bone microenvironment. In conclusion, while acknowledging the complexities surrounding Hif1α function in bone biology, our study provides new insights into its role in melanoma-induced osteocyte ferroptosis. We believe these findings contribute to a deeper understanding of the molecular mechanisms underlying bone pathology associated with cancer metastasis and warrant further investigation into therapeutic strategies targeting the Hif1α/Hmox1 axis.

Given the complexity of melanoma metastasis and the multifaceted nature of cellular interactions within the bone microenvironment, it is important to acknowledge the limitations of our study. While our investigation sheds light on the role of the Hif1α-Hmox1 axis in melanoma-induced osteocyte ferroptosis, it focuses primarily on a subset of molecular pathways and regulatory mechanisms. Consequently, our study may not fully encompass the intricate interplay with other signaling cascades that contribute to melanoma progression and bone metastasis. Furthermore, it should be noted that the study employed a specific melanoma cell line, which may not fully reflect the heterogeneity of melanoma cells observed in clinical settings. While the intracardiac injection model is valuable for studying bone metastasis, it may not fully replicate the natural progression and microenvironmental interactions of melanoma metastasis as seen in patients. It is important to consider these limitations when interpreting the results and exploring potential therapeutic applications. Further validation through preclinical and clinical studies is essential to establish the therapeutic relevance of treatments for melanoma bone metastasis. The discrepancies between the physiological, tumor microenvironment and immune response characteristics of animal models and humans have a considerable impact on the outcomes of preclinical studies. It is possible that human osteocyte responses to ferroptosis-inducing treatments may differ due to species-specific factors. Therefore, further research is required to substantiate the mechanistic insights observed in human patients.

In conclusion, while acknowledging the complexities surrounding Hif1α function in bone biology, our study provides new insights into its role in melanoma-induced osteocyte ferroptosis. We believe these findings contribute to a deeper understanding of the molecular mechanisms underlying bone pathology associated with cancer metastasis and warrant further investigation into therapeutic strategies targeting the Hif1α/Hmox1 axis.

## Materials and methods

### Cell culture

The B16F10 mouse melanoma cells were obtained from the American Type Culture Collection (ATCC) and cultured in DMEM supplemented with 10% fetal bovine serum (FBS) and penicillin-streptomycin under standard conditions of 37 °C and 5% CO2. The MLO-Y4 murine osteocytes were cultured on plates pre-coated with rat tail collagen type I (Corning, Cat. #354236, 0.15 mg/mL), generously provided by Erwin F. Wagner from the Department of Dermatology and Department of Laboratory Medicine, MedUni Wien, Vienna. The cells were cultured in α-MEM supplemented with 2.5% FBS, 2.5% bovine calf serum (BCS, VWR, Cat. #HYCLSH30072.3), and penicillin-streptomycin at 37 °C and 5% CO2, following established protocols.^59^

### Murine osteoclast differentiation

Bone marrow cells from wildtype mice aged six weeks were isolated by flushing the femur and tibia. The cells were plated overnight at 37 °C with 5.5% CO_2_ in a 100 × 20 mm dish in osteoclast medium. This consisted of αMEM and GlutaMAX (Gibco), 10% fetal calf serum (FCS), and 1% penicillin/streptomycin (Gibco), supplemented with 5 ng/mL macrophage colony-stimulating factor (M-CSF) (PeproTech). On the subsequent day, the nonadherent bone marrow macrophages (BMMs) were collected, washed, and subsequently cultured in osteoclast medium with 20 ng/mL M-CSF and 10 ng/mL receptor activator of nuclear factor kappa-B ligand (RANKL) (PeproTech) in 96-well plates (200 μL/well for TRAP staining) or 48-well plates (500 μL/well for RNA analysis) at a concentration of 1 × 10^6^ cells/mL at 37 °C with 5.5% CO2. The medium was replaced every two days. In the experimental design, bone marrow cells treated with M-CSF but without RANKL served as the undifferentiated control group; cells treated with M-CSF and RANKL served as the differentiated experimental group; and cells treated with M-CSF, RANKL, and B16F10 conditioned medium (CM) served as the B16F10-treated experimental group. The fully differentiated osteoclasts (on days 5) were washed with PBS, the cells in 96-well plates were fixed with fixation buffer (MilliporeSigma), and they were stained with TRAP solution. The cells in 48-well plates were prepared for further RNA isolation.

### Mice

Male C57BL/6 N mice, aged six weeks, were obtained from Charles River Laboratories (Sulzfeld, Germany) and housed in the animal facility of the Friedrich-Alexander-Universität Erlangen-Nürnberg Faculty of Medicine. The mice were kept in a controlled environment with a 12-h light and dark cycle at 25 °C.

The injection of B16F10 melanoma cells was performed intracardially by administering 1×10^5^ cells in a 100 μL volume after inducing anesthesia with isoflurane (Abbott; IsoFlo®, Cat. #05260-05), following established protocols. Mice without tumors received PBS injections as controls and were continuously monitored for 24 h.

The treatment group received B16F10 melanoma cells injected as previously described. Subsequently, daily intraperitoneal injections of Ferrostatin-1 (Fer-1, 1 mg/kg) (MedChemExpress, Cat. # HY-100579), Zinc Protoporphyrin (Znpp, 10 mg/kg) (MedChemExpress, Cat. # HY-101193), and Roxadustat (10 mg/kg) (Invivochem, Cat.# V0293-500mg) were administered for 13 consecutive days. The control group received an equal volume of DMSO in PBS.

All mice were euthanized 14 days after B16F10 cell inoculation. The animal experiments followed the approved protocols set by the government of Franconia in Germany (license numbers: mouse models 54-2532.1).

### Bulk RNA sequence

The bulk RNA sequence analysis was conducted by acquiring bone tissues and MLO-Y4 cells as specified in the Figure legends. To ensure enrichment of osteocytes and minimize contamination from other cell types, the bone tissue samples underwent the following treatments before RNA-seq analysis: First, bone tissues were placed in centrifuge tubes containing cold PBS and centrifuged (3 000 *g* for 5 minutes) to remove bone marrow. After bone marrow removal, bone tissues were further processed to eliminate cells attached to the bone surface. The tissues were washed with PBS, gently shaken in PBS containing 1% Triton X-100 for 10 minutes, and then washed several times with PBS to remove surface cells. Finally, the epiphyses of the bones were removed during the process, retaining only the diaphysis for subsequent RNA extraction. This step helps reduce interference from joint cartilage and other non-osteocyte cells.

Next, total RNA was extracted using the QIAGEN RNeasy kit, followed by RNA-seq analysis performed by Novogene in London, UK. The ‘DESeq2′ R package was used for differential gene expression analysis, which enabled the identification of Differentially Expressed Genes (DEGs) between distinct experimental groups.^60^

To annotate biological functions and elucidate enriched pathways associated with these DEGs, we conducted Gene Ontology (GO) functional analysis and KEGG pathway enrichment analysis using the ‘clusterProfiler’ R package.^61^ Furthermore, Gene Set Enrichment Analysis (GSEA) yielded additional information on pertinent biological pathways.^62^ Volcano diagrams were used to generate visual representations of differential gene expression patterns with the ‘ggplot2′ R package.^63^ Additionally, a heatmap was created using the ‘pheatmap’ R package to display the expression profiles across samples.^64^

The data discussed in this publication have been deposited in NCBI’s Gene Expression Omnibus^65^ and are accessible through GEO Series accession number GSE276370 (Bone samples) and GSE276373 (MLO-Y4 samples).

### RNA extraction and real-time quantitative PCR

The cells and bone tissues, treated as described in the Figure legend, were lysed in RNA-Solv (VWR, Cat. #R6830-02) according to the manufacturer’s protocol for RNA extraction. The extracted RNA was then converted into complementary DNA (cDNA) through reverse transcription using the Life Technologies kit (Life Tech, Cat. #4368813) following the provided guidelines.

Quantitative PCR analysis was performed using the Select Master Mix (Applied Biosystems, Life Tech, 447290). The internal control β-Actin was used to normalize the expression levels of target genes (Table 1), ensuring the accuracy and reliability of the analysis.

**Table 1:** Primer list

Primer name	Forward Sequence (5′-3′)	Reverse Sequence (5′-3′)
beta-Actin	TGTCCACCTTCCAGCAGATGT	AGCTCAGTAACAGTCCGCCTAGA
Sclerostin	TGACGCCAAAGATGTGTCCGAG	CACCACTTCACGCGCCCGAT
Runx2	TCGGAGAGGTACCAGATGGG	TGAAACTCTTGCCTCGTCCG
Phex	CTGGCTGTAAGGGAAGACTTCC	GCTCCTAAAAGCACAGCAGTGTC
Pdpn	ACAACCACAGGTGCTACTGGAG	GTTGCTGAGGTGGACAGTTCCT
Hmox1	CACTCTGGAGATGACACCTGAG	GTGTTCCTCTGTCAGCATCACC
HIF1a	CCTGCACTGAATCAAGAGGTTGC	CCATCAGAAGGACTTGCTGGCT
Gpx4	CTGGGAAATGCCATCAAAT	GTCCTTCTCTATCACCTGG
Ftl1	CCTCGAGTTTCAGAACGATCGC	CCTGATTCAGGTTCTTCTCCATG
Fth1	GCCGAGAAACTGATGAAGCTGC	GCACACTCCATTGCATTCAGCC
Dmp1	CCACCACCCACGAACAGTGAGT	CATCCTCCTTATCGGCGCCGG
Dkk1	ATCTGTCTGGCTTGCCGAAAGC	GAGGAAAATGGCTGTGGTCAGAG
Col1a1	CGACCTCAAGATGTGCCACT	TCCGTACTCGAACGGGAATC
Bax	AGGATGCGTCCACCAAGAAGCT	TCCGTGTCCACGTCAGCAATCA
Caspase 3	GGAGTCTGACTGGAAAGCCGAA	CTTCTGGCAAGCCATCTCCTCA
Caspase 8	ATGGCTACGGTGAAGAACTGCG	TAGTTCACGCCAGTCAGGATGC

### Terminal Deoxynucleotidyl Transferase-mediated dUTP Nick End Labeling Staining (TUNEL staining)

TUNEL staining was performed on MLO-Y4 cells and bone tissues following specific guidelines outlined in the figure legend. To quantify osteocyte death percentages, both in vivo and in vitro, the One-step TUNEL In Situ Apoptosis Kit (biomol, Cat# E-CK-A320) protocol was followed as per the manufacturer’s instructions.^66^

### PI/Annexin V staining

MLO-Y4 cells exposed to B16F10-derived conditioned medium, with or without specific inhibitors as described in the figure legends, were stained using Annexin V in Annexin V binding buffer (BioLegend, Cat# 422201) at a 1:200 dilution.^67^ This staining process was performed in the dark at room temperature for 20 minutes. The cells were then washed twice with PBS and stained with a 1:1 000 dilution of propidium iodide (PI) (Thermo Fisher Scientific, Cat# 00-6990-50) for 5 minutes. Flow cytometry was used to analyse the stained cells, and the data obtained were processed and analysed using FlowJo software, version 10.4.

### Ferroptosis probes

MLO-Y4 cells exposed to B16F10-derived conditioned medium, with or without specific inhibitors as described in the figure legends, were assessed for ferroptosis using C11-BODIPY 581/591 fluorescent sensors^68^ (Invitrogen, Cat# D3861) and H2DCFDA^69^ (MedChemExpress, Cat# HY-D0940). After treatment, cells were washed twice with PBS and incubated with 2 μmol/L C11-BODIPY 581/591 and H2DCFDA for 30 minutes in the dark. The cells were then washed with PBS and the stained cells were analysed by flow cytometry. Data were analysed using FlowJo software (version 10.4).

### Cell Transfection and Lentiviral Infection

Specific shRNAs against HMOX1 (shHMOX1) and negative control (shNC) were obtained from VectorBuilder (Cat# No. Ecoli(VB900046-5163hfx) for shRNA1; Ecoli(VB900046-5169gxz) for shRNA2; Ecoli(VB900046-5173muu) for shRNA3; Ecoli(VB010000-0009mxc)-P for negative control. HIF1α overexpression plasmid (pcDNA3 mHIF-1α, addgene, Cat# 44028), pcDNA3 was performed as an empty vector (addgene, Cat# 13032). Specific shRNAs against HIF1α (shHIF1α) and negative control (shNC) were obtained from VectorBuilder (pLV[shRNA]-mCherry>mHif1a[shRNA#1]; Cat# VB900122-2420djn) for shRNA1; pLV[shRNA]-mCherry>mHif1a[shRNA#2] (Cat# VB900122-2421ngn) for shRNA2; mCherry lentiviral control vector pLV-mCherry (Cat# VB010000-9390nka-P) for negative control. Lentiviral transfer plasmids were co-transfected into HEK293T cells using a Lipofectamine 3000 transfection reagent. Cell culture media containing lentiviral particles were collected 48 h after transfection, filtered through a 0.45 μm syringe filter and concentrated by ultracentrifugation at 12 000 r/min for 1 h. The viral pellet was resuspended in phosphate-buffered saline (PBS) and stored at -80 °C until further use.

MLO-Y4 cells were cultured in appropriate media. For lentiviral infection, cells were plated at the desired density and exposed to lentiviral vectors. Polybrene (Sigma, Cat# TR-1003-G) (5 μg/mL) was added to increase transduction efficiency. Cells were incubated with lentivirus for 48 h and then maintained in fresh culture media. Puromycin (Invivogen, Cat# ant-pr-1) (5 μg/mL) was used to sort the infected cells. Western blotting was performed to further determine the knockdown efficiency.

### Chromatin immunoprecipitation

ChIP experiments were performed using ChIP-IT Express Kit (Active motif, Cat# 53040). Cells were sonicated at 40% power for 15 cycles of 4 pulses (20 sec with 30 sec breaks on ice between each pulse). The following reaction components were used: Protein G Magnetic Beads 25 μL, ChIP buffer 110 μL, sheared chromatin 60 μL, PIC 3 μL, anti-HIF1α (Novus, Cat# NB100-105) 2 μL. On the second day, magnetic beads were washed and chromatin was eluted for qPCR (Table 2). Sheared chromatin was used as input to normalize DNA loading.

**Table 2:** Chip primer list

Primer name	Forward Sequence (5′-3′)	Reverse Sequence (5′-3′)
HRE1	TGACCCGCGTACTTAAAGGG	GGTTCTGCTCGATTCAGGCT
HRE2	CCAGTCGCCTCCAGAGTTTC	GATTCAGGCTCCGGGCTATG
HRE3	GCGTACTTAAAGGGCTGGC	TCCACTCACTGGTTGTATGCG

### Dual-Luciferase Assay

#### Plasmid construction

The wild-type (WT) and mutant (MUT) Hmox1 promoter regions were cloned into the pGL4.10 vector using BglII and HindIII restriction sites. The following plasmids were constructed: pGL4.10 (Empty Vector); pGL4.10-Hmox1 promoter (WT); pGL4.10-Hmox1 promoter (MUT).

#### Cell culture and transfection

HEK 293 T cells were cultured in DMEM supplemented with 10% FBS and 1% penicillin-streptomycin at 37 °C in a 5% CO2 incubator. For transfection, cells were seeded into 96-well plates and incubated overnight. The cells were divided into six groups for transfection: 1. pGL4.10 + pcDNA3.1( + )-MSC-6xHis + pRL-CMV (Empty Vector Control); 2. pGL4.10 + pcDNA3.1(+)-Hif1α-6xHis + pRL-CMV (HIF1α Overexpression Control); 3. pGL4.10-Hmox1 promoter (WT) + pcDNA3.1( + )-MSC-6xHis + pRL-CMV (WT Promoter + Empty Vector); 4. pGL4.10-Hmox1 promoter (WT) + pcDNA3.1(+)-Hif1α-6xHis + pRL-CMV (WT Promoter + HIF1α Overexpression); 5. pGL4.10-Hmox1 promoter (MUT) + pcDNA3.1( + )-MSC-6xHis + pRL-CMV (MUT Promoter + Empty Vector); 6. pGL4.10-Hmox1 promoter (MUT) + pcDNA3.1(+)-Hif1α-6xHis + pRL-CMV (MUT Promoter + HIF1α Overexpression). Each well was transfected with a total of 1 μg DNA using Lipo8000™ Transfection Reagent. The DNA mixture was gently mixed with the transfection reagent according to the manufacturer’s protocol and added directly to the cells. The cells were incubated for 30 h post-transfection.

#### Dual-Luciferase Assay

After 30 h of transfection, the dual-luciferase reporter assay was performed using the Dual Luciferase Reporter Gene Assay Kit (Beyotime RG027). Firefly and Renilla luciferase activities were measured sequentially using a luminometer. Firefly luciferase activity was normalized to Renilla luciferase activity to control for transfection efficiency.

### Immunofluorescence

Immunofluorescence staining of bone tissue involves incubation of sections or cell slides with specific primary antibodies, including anti-HIF1α (Cayman Chemicals, Cat# 10006421-1), anti-HIF2α (Novus, Cat#NB100-122), anti-HMOX1 (Proteintech, Cat# 10701-1-AP) and anti-GPX4 (Proteintech, Cat# 67763-1-Ig), all the first antibodies were diluted at a concentration of 1:300 in 2% BSA in PBS. This was followed by incubation with DyLight 594-conjugated (1:200, Vector, Cat. #Dl-1094) or Alexa Fluor 488-conjugated secondary antibodies (1:200, Vector, Cat. #Dl-2488-1.5). Sections or cell slides were mounted with fresh DAPI (Biozol, Cat# H-1200) and imaged using a Keyence fluorescence microscope. Image quantification was performed using ImageJ.

For cell immunofluorescence staining, cells were seeded on 12 mm circular coverslips and treated with anti-HMOX1, anti-FTL1 (Proteintech, Cat# 10727-1-AP) and anti-LC3 (Proteintech, Cat# 14600-1-AP) antibodies, all the first antibodies were diluted at a concentration of 1:300 in 2% BSA in PBS. This was followed by incubation with DyLight 594-conjugated (1:200, Vector, Cat. #Dl-1094) or Alexa Fluor 488-conjugated secondary antibodies (1:200, Vector, Cat. #Dl-2488-1.5). Cells were then mounted with fresh DAPI and imaged using a Keyence fluorescence microscope and a confocal laser scanning fluorescence microscope (LSM700; Zeiss). Quantitative analysis of the images obtained was performed using ImageJ.

### Western blot

For Western blot analysis, cells subjected to the treatments indicated in the figure legend were lysed on ice using RIPA buffer containing protease and phosphatase inhibitors for 30 minutes. Lysates were centrifuged at 12 000 *g* for 10 minutes at 4 °C. BCA normalization was performed to ensure equal loading of the cell lysates. Equal amounts of protein were then loaded and separated on SDS-PAGE gels, followed by transfer to 0.2 μm PVDF membranes (Thermo Scientific, Cat. No. 88520). The membranes were incubated with primary antibodies including anti-HIF1α, anti-HIF2α, anti-HMOX1, anti-GPX4, anti-FTL1 and anti-LC3, all the antibodies were diluted at a concentration of 1:1 000 in TBS-T buffer (Tris-buffered saline with 0.1% Tween20). Quantification of protein bands was performed using ImageJ.

### μCT analysis

Long bones were fixed in 4% PFA overnight prior to assessment. All proximal tibial metaphyses were scanned using the SCANCO Medical μCT 40 cone beam desktop micro computer tomograph. Settings were optimized to visualize calcified tissue at 55 kVp with a current of 145 μA and 200 ms integration time for 500 projections/180°.

An isotropic voxel size of 8.4 μm was used for 3D volume segmentation. Customized greyscale thresholds (threshold in the white region >100) were applied using the Open VMS operating system (SCANCO Medical). Bone structure analysis focused on the proximal tibial metaphysis, starting 0.43–0.42 mm from a defined landmark in the growth plate and extending 1.68 mm distally (200 tomograms).

To assess bone structure within fractured femoral calluses, the callus center was identified and bone volume (BV) was measured over a 2.10 mm span (250 tomograms).

### Transmission electron microscopy

For transmission electron microscopy (TEM), MLO-Y4 cells were prepared as described in the figure legends. The cells were harvested and initially fixed with 2.5% glutaraldehyde in cacodylate buffer (pH 7.4, containing sucrose). After washing, the cells were post-fixed with 2% osmium tetroxide in cacodylate buffer and subsequently embedded in 2% agarose. During ethanol dehydration, the cells were treated with 1.0% uranyl acetate in 70% ethanol. The samples were then embedded in Araldite and sectioned using an ultramicrotome (Reichert Ultracut, Vienna, Austria). Image acquisition was performed with a Zeiss EM10 electron microscope (Carl Zeiss AG) equipped with a Gatan SC1000 Orius™ CCD camera, utilizing Digital Micrograph™ software (Gatan GmbH, Munich, Germany).

### Histological analysis

Histological analysis was performed on serial paraffin sections. An H&E staining kit (Carl Roth) was used to assess general morphology. For lung tissue, PAS staining was employed to further clarify tumor progression. Histomorphometric evaluation was performed using a Zeiss Axioskop 2 microscope (Carl Zeiss) equipped with the OsteoMeasure image analysis system (Osteometrics). Hematoxylin and eosin-stained sections were used specifically to assess the degree of tumor invasion and the rate of osteocyte death. The Leukocyte Acid Phosphatase Kit (Sigma) was used to detect osteoclasts. All histological analyses were performed using a Zeiss Axioskop 2 microscope (Carl Zeiss), equipped with a digital camera and the OsteoMeasure image analysis system (Osteometrics).

### Statistical analysis

Statistical analyses were performed with GraphPad Prism v9. Two-tailed Student’s t-tests compared two groups, while one-way or two-way ANOVA evaluated multiple groups. Pearson’s correlation test was used for correlation analysis, which was assessed by linear regression F-test. A significance threshold of *P* < 0.05 was used to indicate statistical significance.

## Supplementary information

Supplementary Figure 1

Supplementary Figure 2

Supplementary Figure 3

Supplementary Figure 4

Supplementary Figure 5

Supplementary Figure 6

Supplementary Figure 7

Supplementary Figure 8

Supplementary Figure Legends

## Data Availability

Data and materials will be made available upon request and, if applicable, material transfer agreements.
