# Supplementary figures and images for: Melanoma bone metastasis-induced osteocyte ferroptosis via the HIF1α-HMOX1 axis
Source: Bone Res. 2025 Jan 16;13:9. doi: 10.1038/s41413-024-00384-y (PMC11735842; doi:10.1038/s41413-024-00384-y)

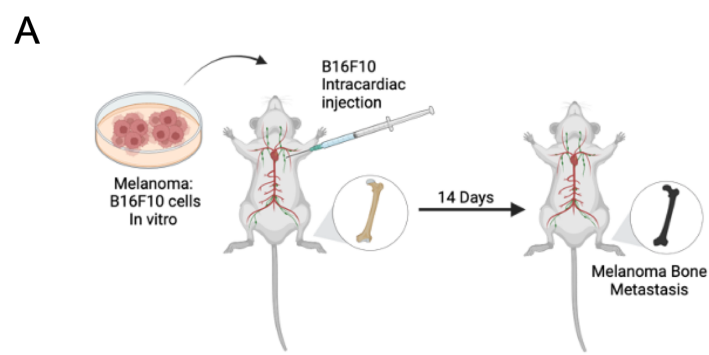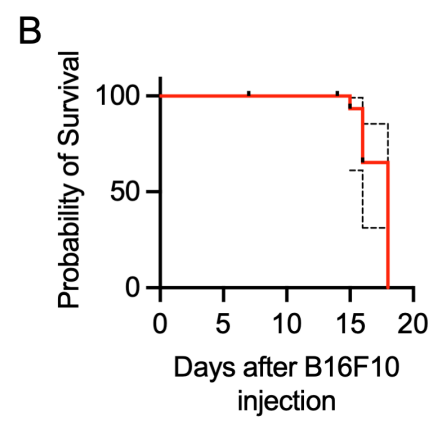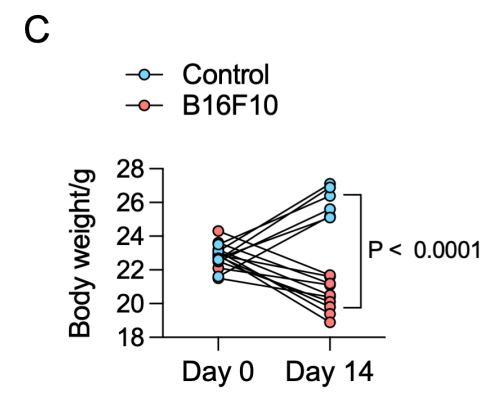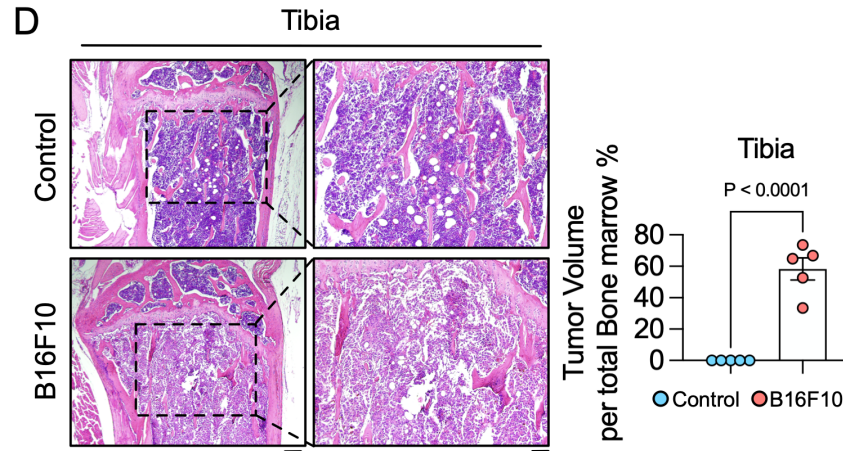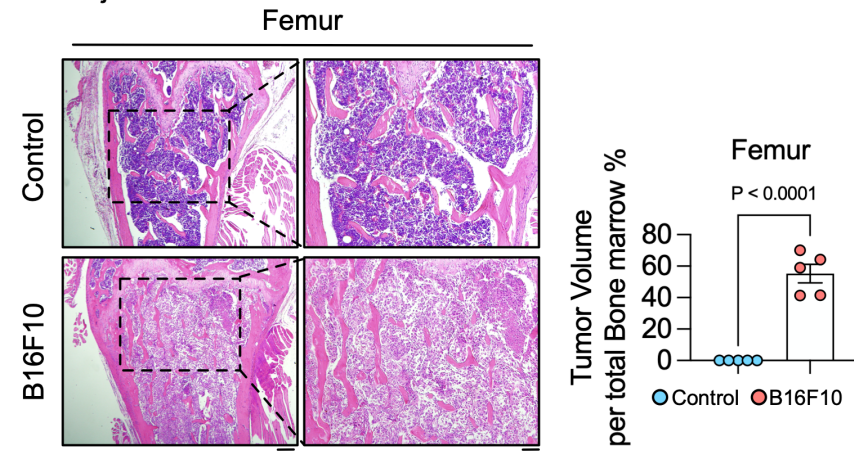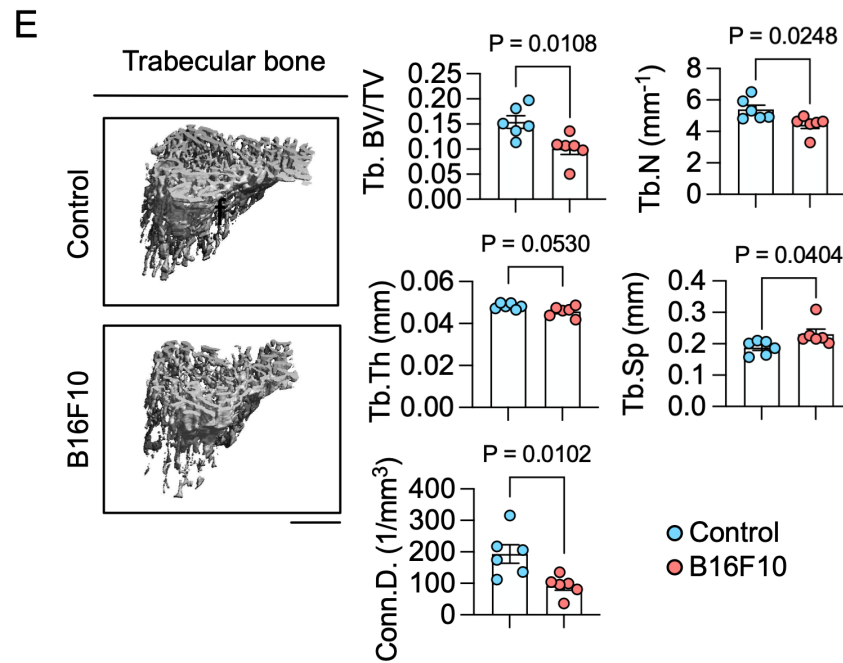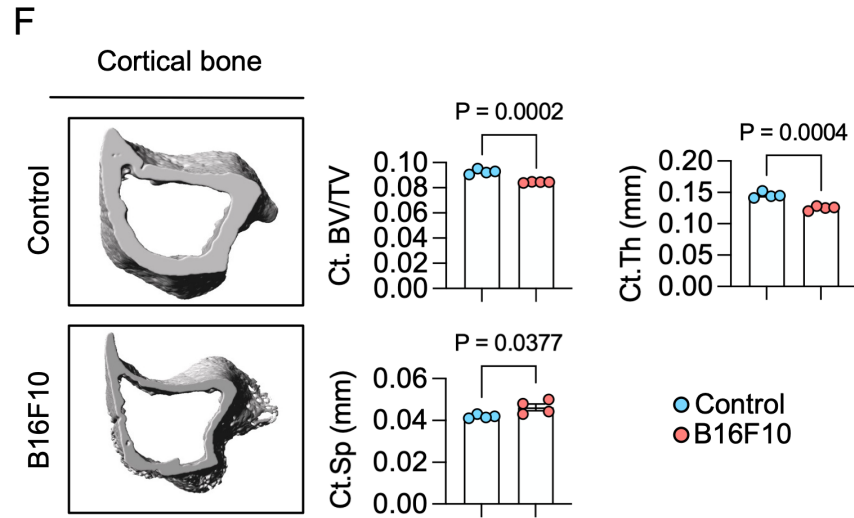

Supplement: Supplementary file 1 — Supplementary Figure 1 [file 41413_2024_384_MOESM1_ESM.pdf]

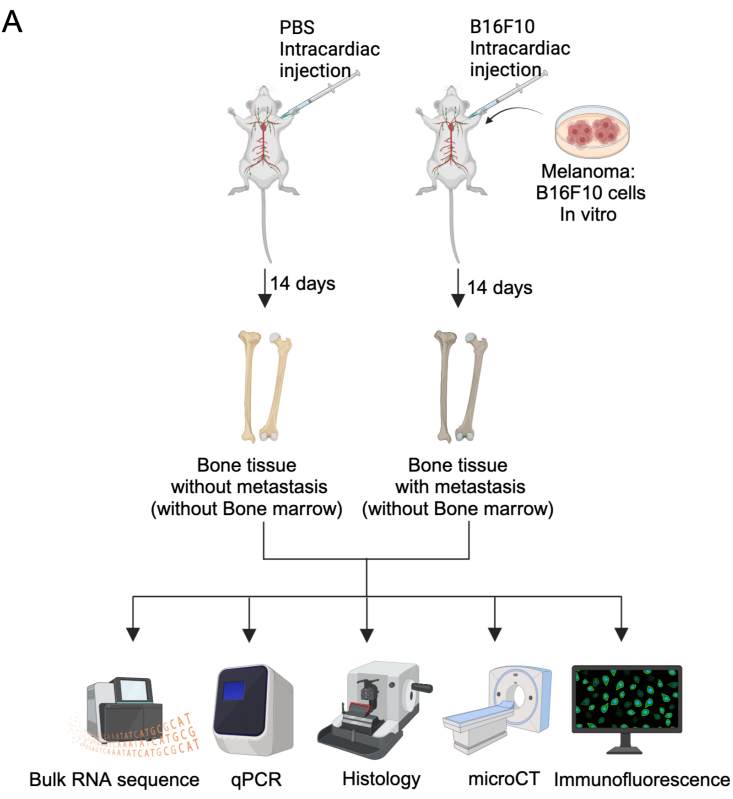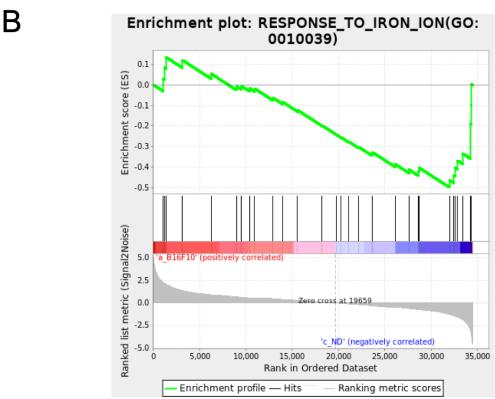

B16F10 metastasis bone vs normal bone

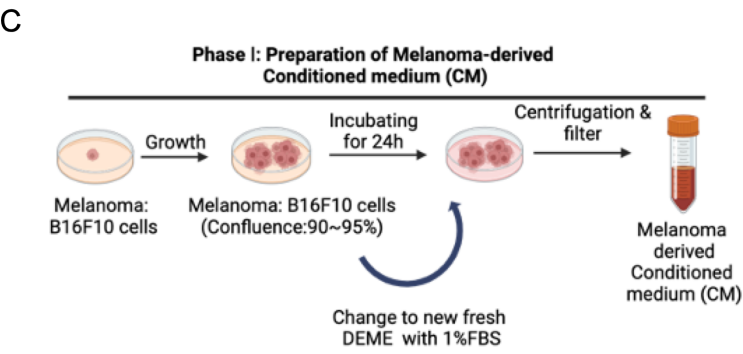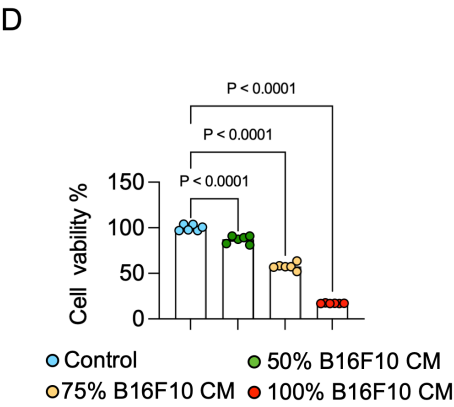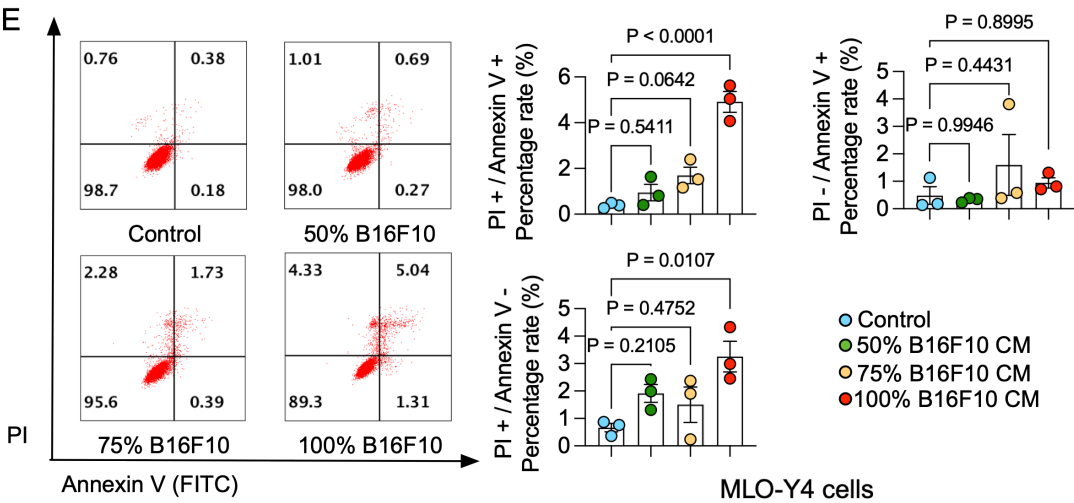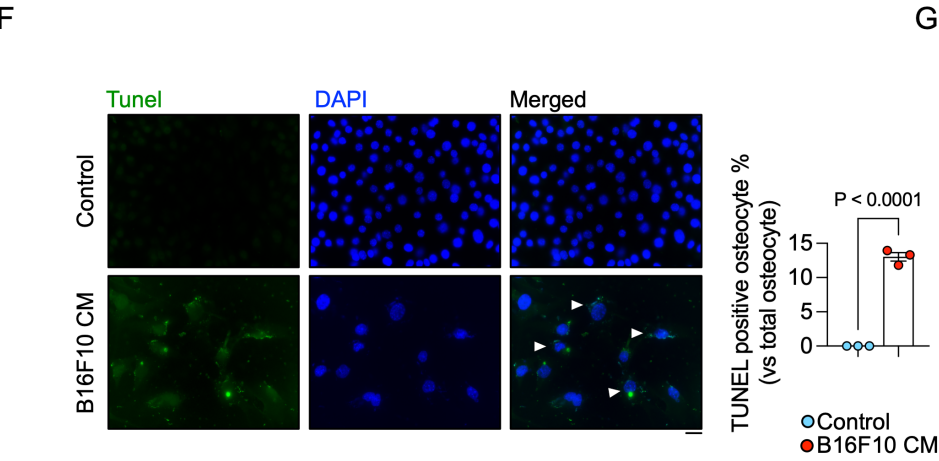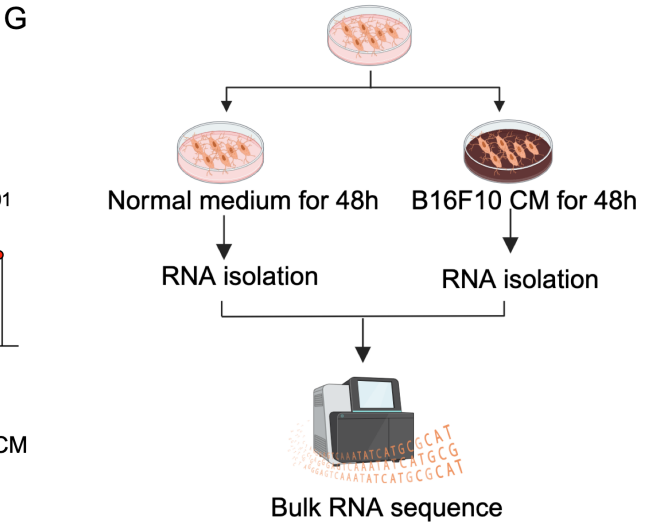

Supplement: Supplementary file 2 — Supplementary Figure 2 [file 41413_2024_384_MOESM2_ESM.pdf]

A

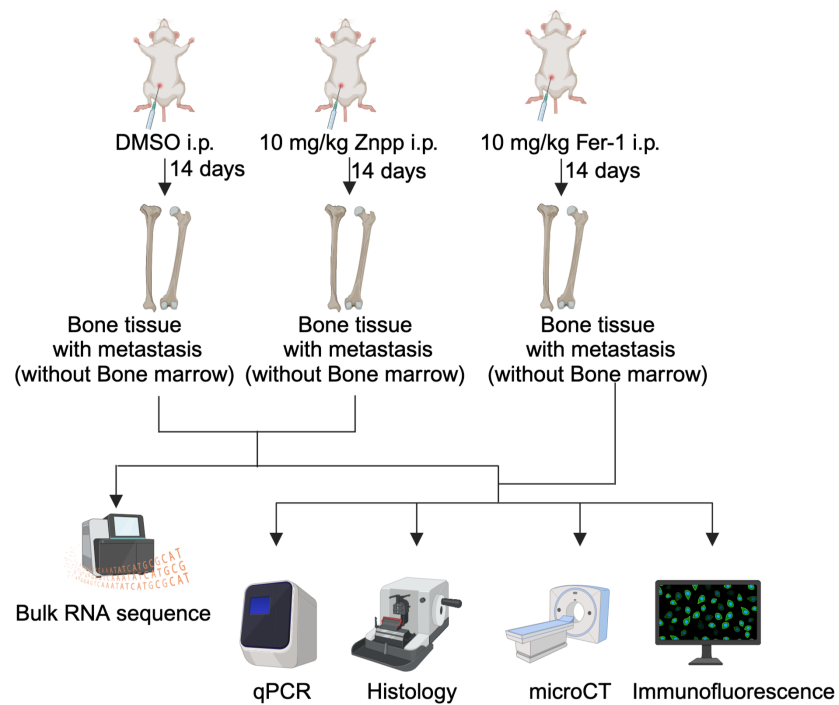

B

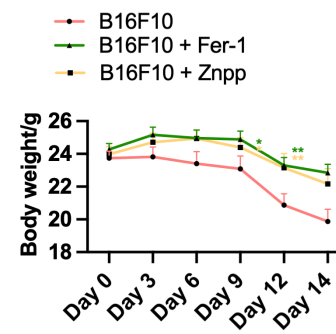

C

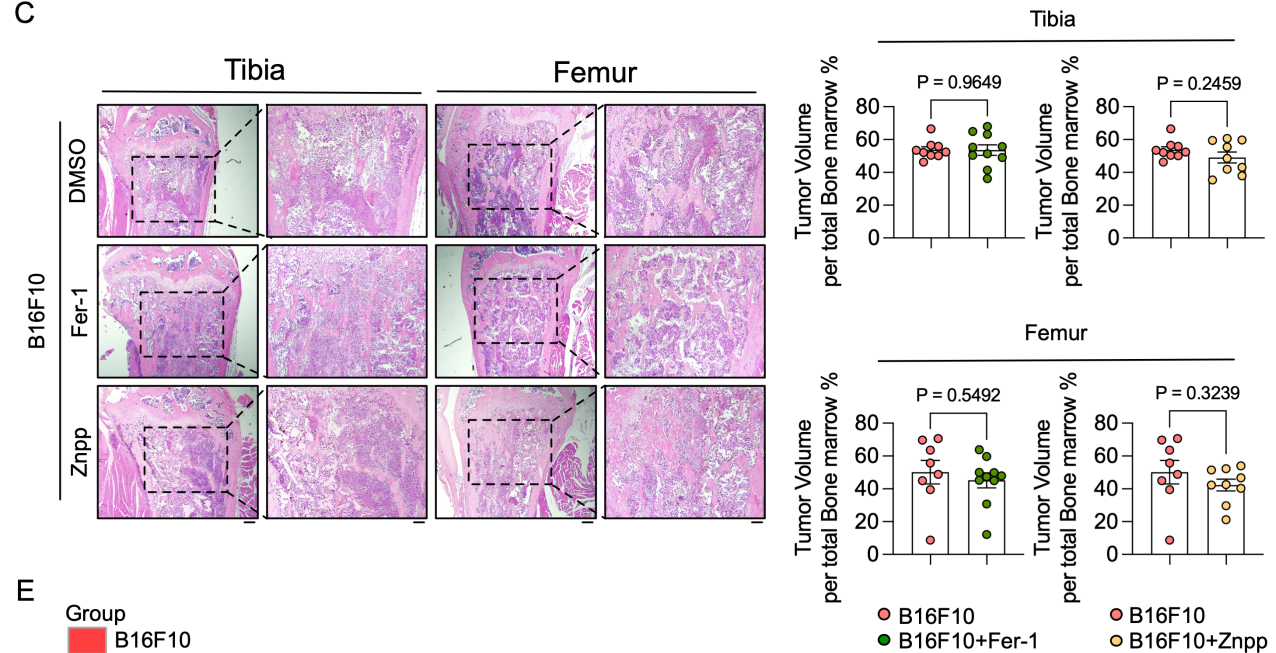

D

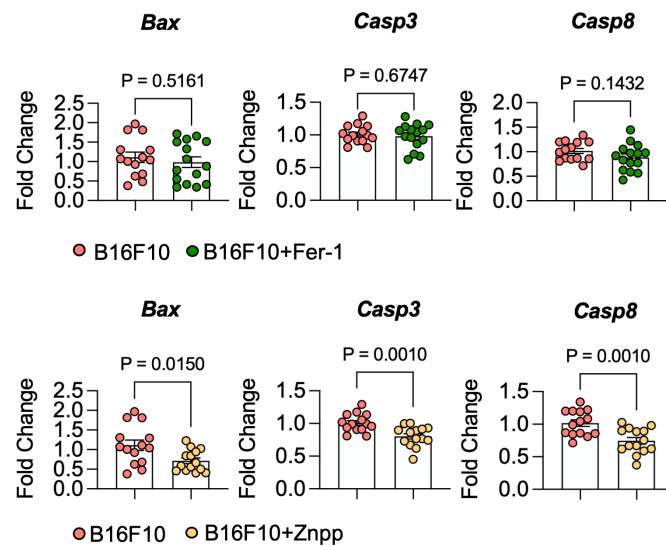

E

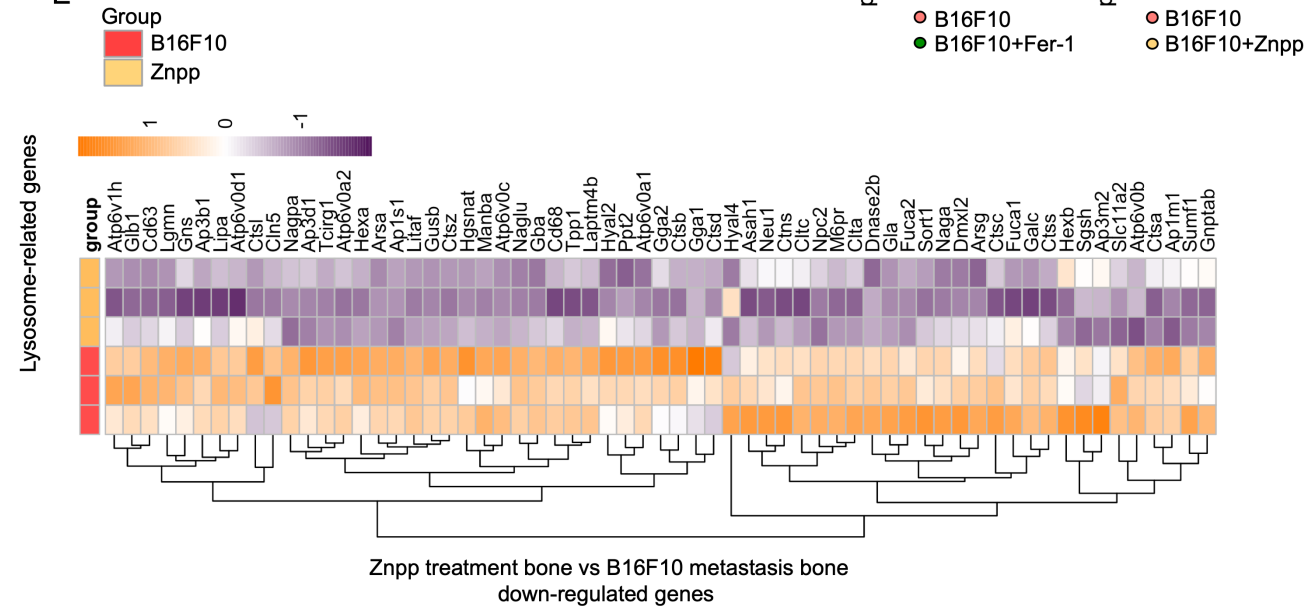

Supplement: Supplementary file 3 — Supplementary Figure 3 [file 41413_2024_384_MOESM3_ESM.pdf]

A

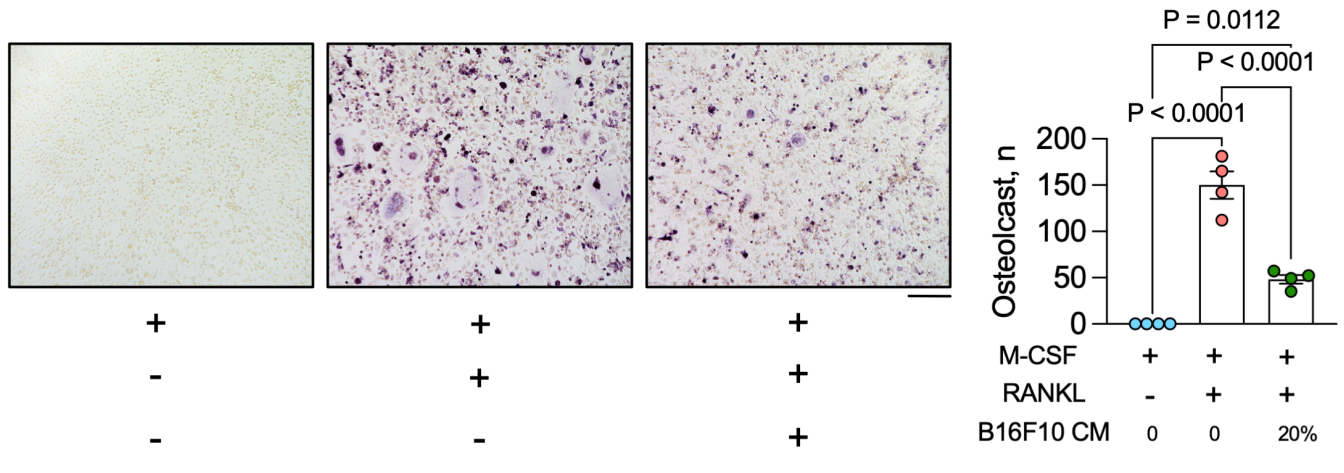

B

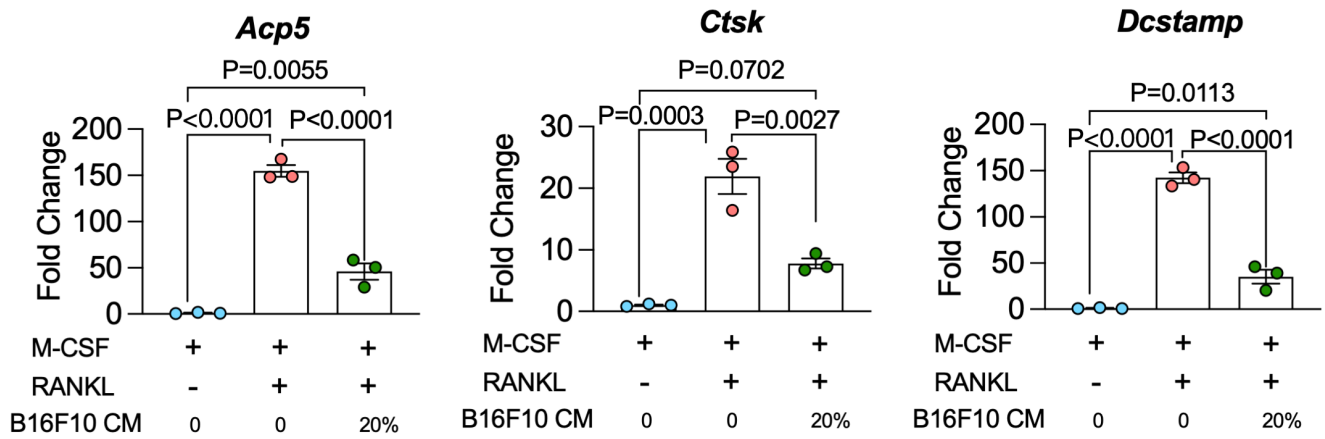

C

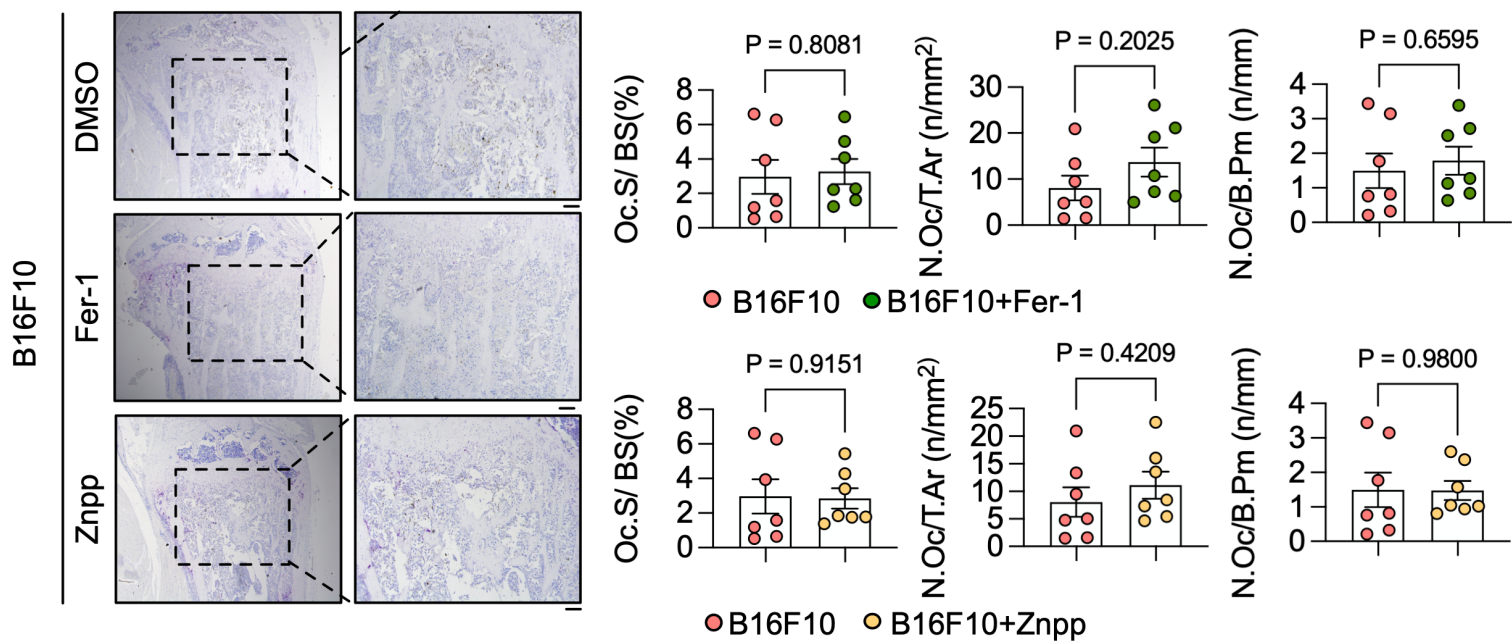

Supplement: Supplementary file 4 — Supplementary Figure 4 [file 41413_2024_384_MOESM4_ESM.pdf]

A

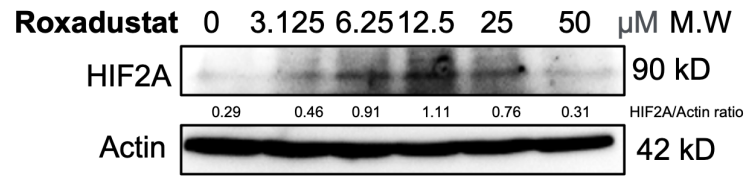

B

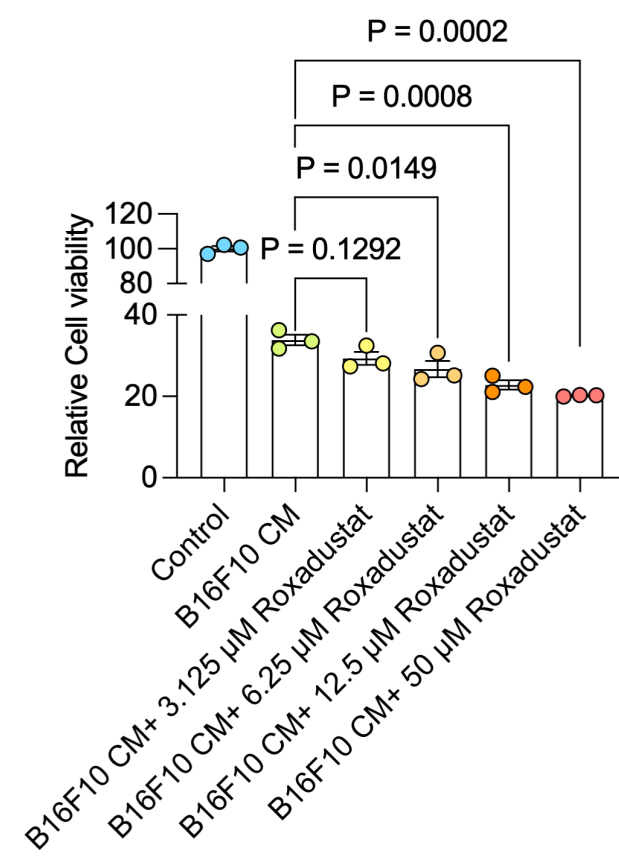

C

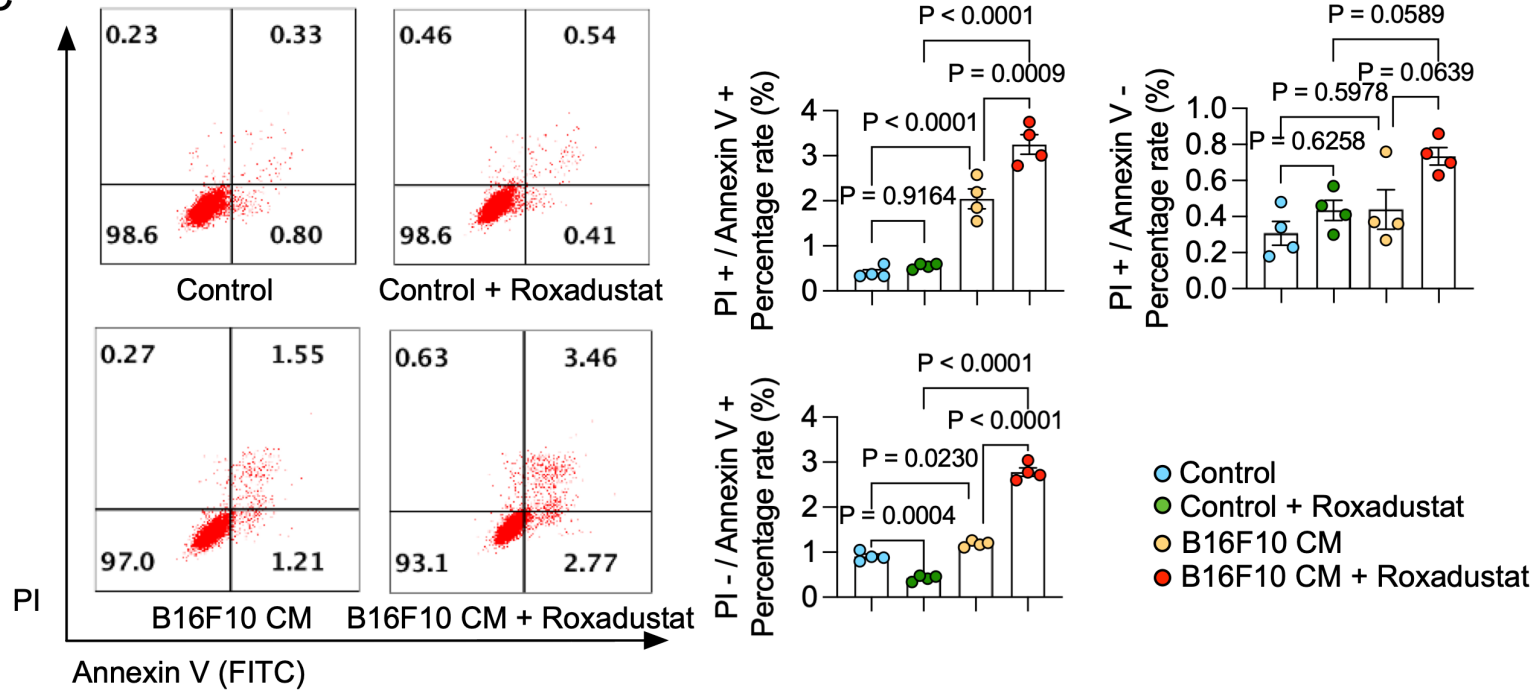

Supplement: Supplementary file 5 — Supplementary Figure 5 [file 41413_2024_384_MOESM5_ESM.pdf]

**A**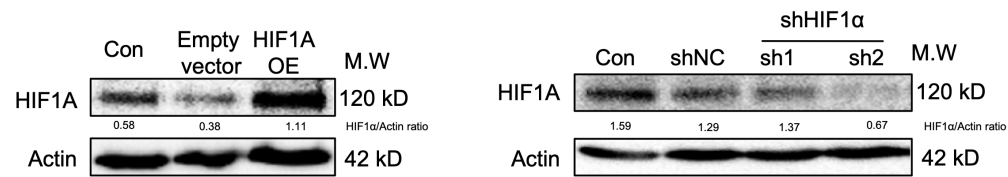**B**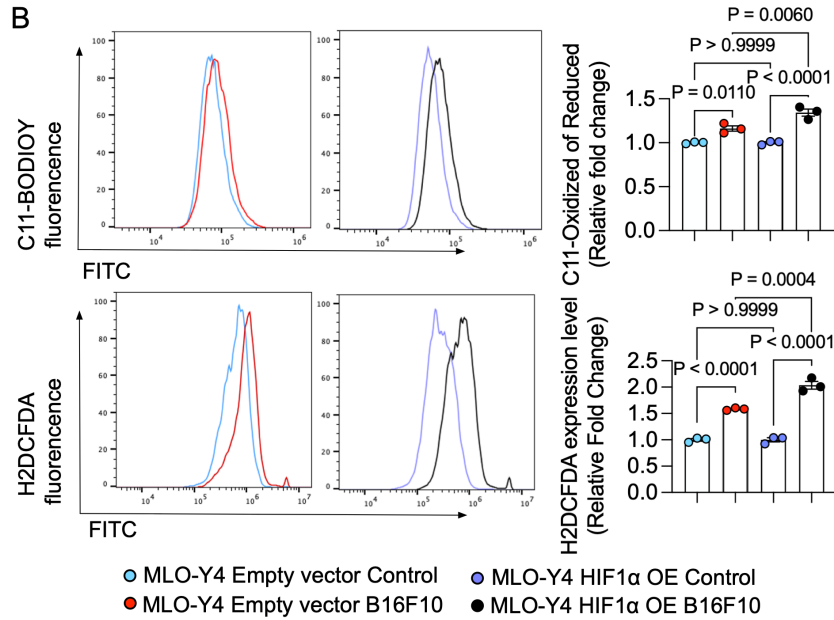**C**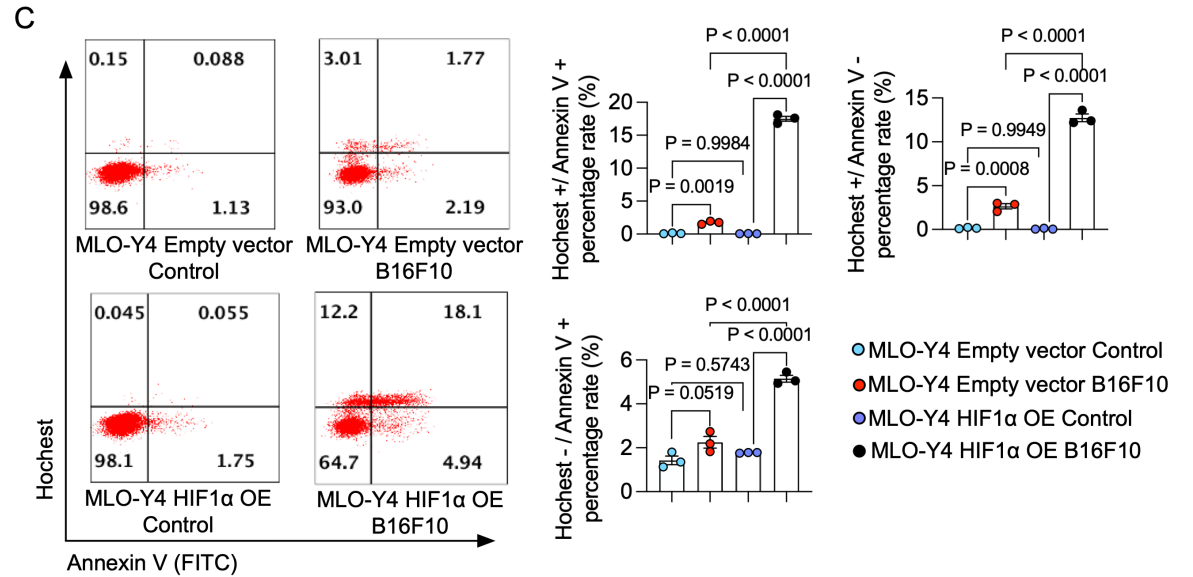**D**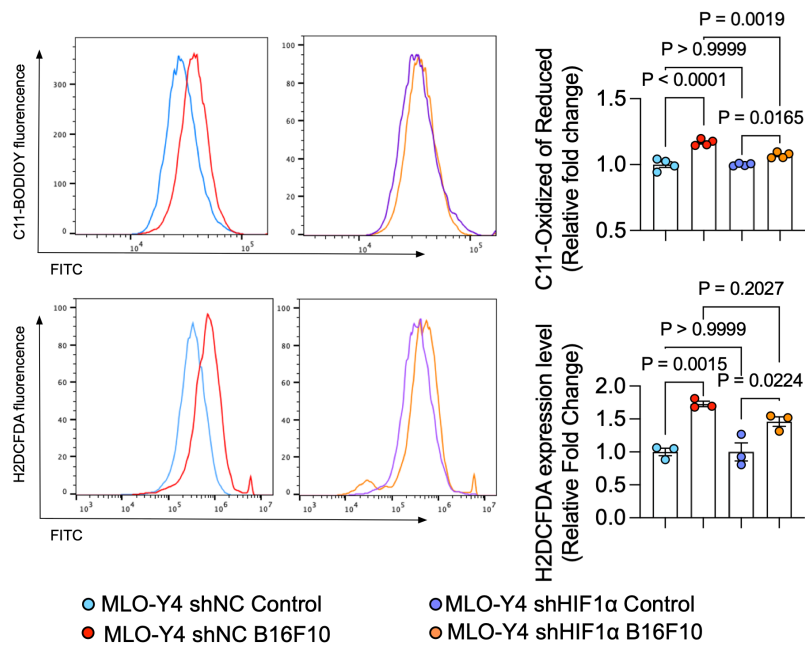**E**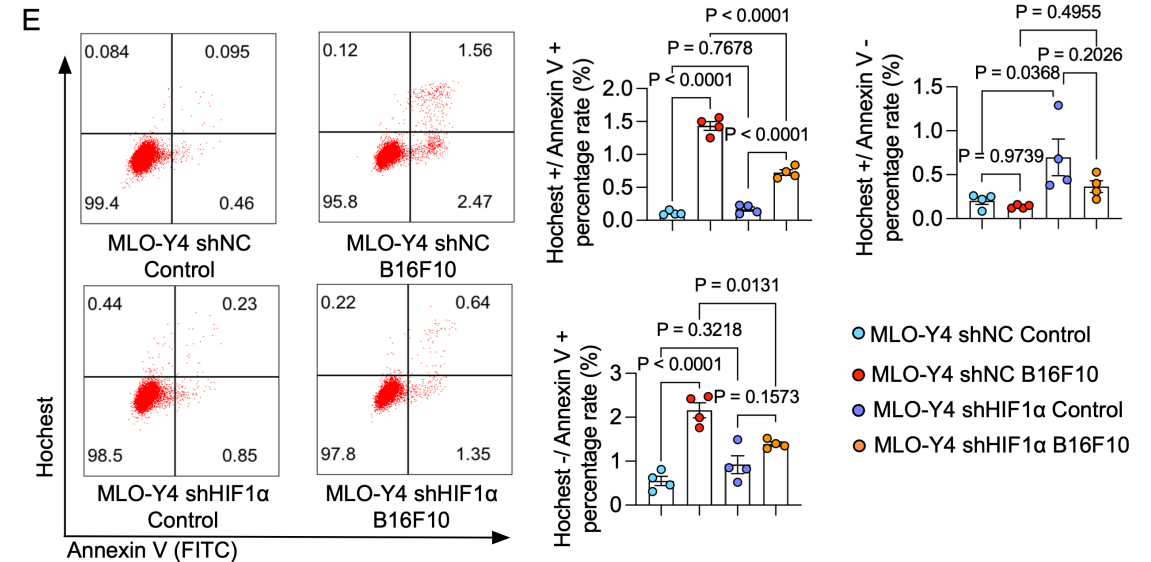

Supplement: Supplementary file 6 — Supplementary Figure 6 [file 41413_2024_384_MOESM6_ESM.pdf]

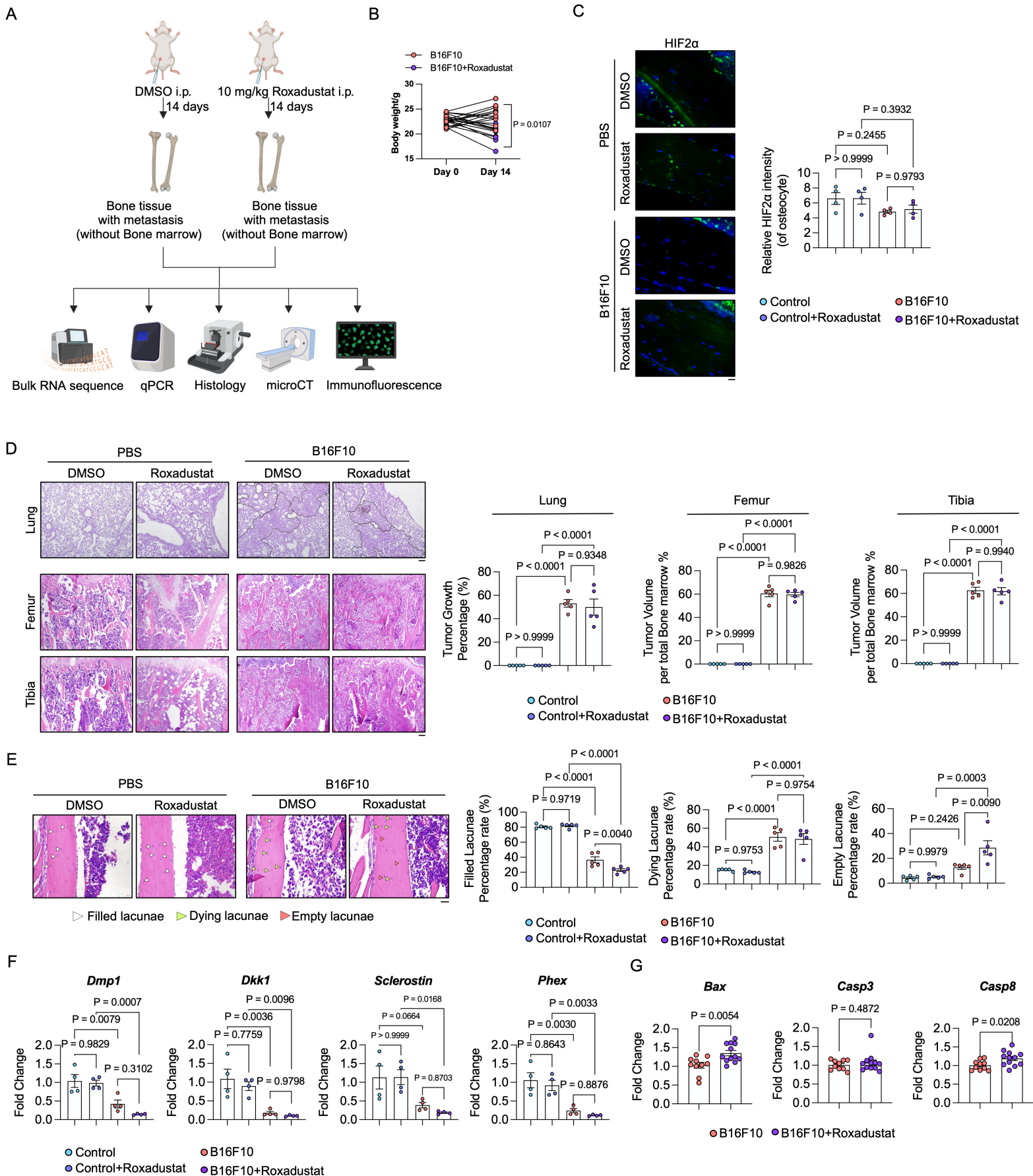

Supplement: Supplementary file 7 — Supplementary Figure 7 [file 41413_2024_384_MOESM7_ESM.pdf]
