# Supplementary material for: Melanoma bone metastasis-induced osteocyte ferroptosis via the HIF1α-HMOX1 axis
Source: Bone Res. 2025 Jan 16;13:9. doi: 10.1038/s41413-024-00384-y (PMC11735842; doi:10.1038/s41413-024-00384-y)
Supplement: Supplementary file 9 — Supplementary Figure Legends [file 41413_2024_384_MOESM9_ESM.docx]

**Supplementary Figure 1.**

**A.** Schematic of the melanoma bone metastasis model via intracardiac injection of B16F10 cells.

**B.** Survival analysis of mice following intracardiac injection of B16F10 cells (n=5).

**C.** Body weight changes in control mice and mice after B16F10 cell injection (n ≥ 6 per group).

**D.** H&E staining of proximal tibia and femur sections from control mice or 14 days post-B16F10 injection. Tumor-invaded area quantified using ImageJ. Scale bars: 200 μm (left), 100 μm (right).

**E.** µCT analysis of trabecular bone from control and B16F10-injected mice, showing trabecular bone volume/total volume (Tb.BV/TV), trabecular separation (Tb.Sp), number (Tb.N), thickness (Tb.Th), and connective density (n=4).

**F.** µCT analysis of cortical bone from control and B16F10-injected mice, showing cortical bone volume/total volume (Ct.BV/TV), separation (Ct.Sp), and thickness (Ct.Th) (n=4).

Statistical significance was determined by a 2-tailed Student’s t-test.

**Supplementary Figure 2**

**A.** Schematic of experimental design and procedures for bone preparation for Bulk RNA sequencing, qPCR, histology, µCT, and immunofluorescence.

**B.** GSEA showing enrichment of the 'RESPONSE_TO_IRON_ION' pathway in B16F10 group.

**C.** Diagram of the preparation of melanoma cell-derived conditioned medium (CM).

**D.** MLO-Y4 cell viability after 48 hours treatment with 0, 50%, 75%, and 100% B16F10 CM, assessed by CCK-8 assay.

**E.** Quantification of MLO-Y4 cell death after 48 hours treatment with 0, 50%, 75%, and 100% B16F10 CM, evaluated via annexin-V and PI staining by flow cytometry.

**F.** TUNEL staining of MLO-Y4 cells treated with or without 75% B16F10 CM for 48 hours. Nuclei are visualized with DAPI (blue); white arrows indicate TUNEL-positive cells. Scale bars: 20 μm.

**G.** Schematic of experimental design and procedure for MLO-Y4 preparation for RNA sequencing.

Statistical significance was determined by a 2-tailed Student’s t-test for single comparisons (F) and one-way ANOVA for multiple comparisons (D, E).

**Supplementary Figure 3**

**A.** Schematic of experimental design and procedures for bone preparation for Bulk RNA sequencing, qPCR, histology, microCT, and immunofluorescence.

**B.** Body weight changes in mice injected with B16F10 and treated with DMSO, 1 mg/kg Fer-1, or 10 mg/kg Znpp by intraperitoneal injection (i.p.).

**C.** Hematoxylin and eosin (H&E) staining of proximal tibia and femurs from mice described in (B). Tumor-invaded areas quantified using ImageJ.

**D.** mRNA expression of Bax, Caspase 3, and Caspase 8 in long bone (without bone marrow) from mice described in (B). (n ≥ 8 per group).

**E.** Heatmap of lysosome pathway-associated DEGs among the aforementioned groups.

Statistical significance was determined by a 2-tailed Student’s t-test for single comparisons.

**Supplementary Figure 4**

**A.** TRAP+ osteoclasts quantified and representative TRAP staining images of mature osteoclasts at day 5 for the undifferentiation group (no RANKL, no B16F10 CM), differentiation group (RANKL only), and B16F10 CM-treated group (RANKL + 20% B16F10 CM). Scale bar: 200 μm.

**B.** mRNA expression of Acp5, Ctsk, and Dcstamp in mature osteoclasts at day 5 for the groups as mentioned in A.

**C.** Representative TRAP staining images and quantification of osteoclast surface/bone surface (Oc.S/BS), osteoclast number/tissue area (N.Oc/T.Ar), and osteoclast number/bone perimeter (N.Oc/B.Pm.) from mice injected with B16F10 and treated with DMSO, 1 mg/kg Fer-1, or 10 mg/kg Znpp (n≥6 per group).

Statistical significance was assessed using a 2-tailed Student’s t-test (C) or one-way ANOVA (A, B).

**Supplementary Figure 5**

**A.** Western blot of HIF2α in MLO-Y4 cells treated with increasing doses of Roxadustat for 48 hours. Actin was used as an internal control.

**B.** Cell viability of MLO-Y4 cells with or without 75% B16F10-derived CM, treated with various Roxadustat concentrations for 48 hours, assessed by CCK-8 assay.

**C.** Cell death in MLO-Y4 cells with or without 75% B16F10-derived CM, treated with or without Roxadustat, evaluated by Annexin V and PI staining followed by flow cytometry.

Statistical significance was determined by one-way ANOVA.

**Supplementary Figure 6**

**A.** Western blot of HIF1α in MLO-Y4 cells with empty vector or HIF1α overexpression plasmid (*left*) and with scrambled shRNA (shNC) or HIF1α-targeting shRNA (shHIF1α) (*right*), using actin as a control.

**B.** FACS analysis of ferroptosis in MLO-Y4 cells (empty vector or HIF1α overexpression), treated with or without 75% B16F10-derived CM for 48 hours, using C11 BODIPY 581/591 and H2DCFDA.

**C.** Cell death assessment in MLO-Y4 cells (empty vector or HIF1α overexpression) treated with or without 75% B16F10-derived CM for 48 hours, via Annexin V and Hoechst staining.

**D.** FACS analysis of ferroptosis in MLO-Y4 cells (shNC or shHIF1α), treated with or without 75% B16F10-derived CM for 48 hours, using C11 BODIPY 581/591 and H2DCFDA.

**E.** Cell death assessment in MLO-Y4 cells (shNC or shHIF1α), treated with or without B16F10-derived CM for 48 hours, via Annexin V and Hoechst staining.

Statistical significance was assessed using a one-way ANOVA.

**Supplementary Figure 7**

**A.** Schematic of experimental design and procedures for bone preparation for Bulk RNA sequencing, qPCR, histology, microCT, and immunofluorescence.

**B.** Body weight changes in B16F10 metastasis mice with or without daily 10 mg/kg Roxadustat (n=5 per group).

**C.** HIF2α immunofluorescence in proximal tibia of normal and B16F10 metastasis mice, with or without daily 10 mg/kg Roxadustat. Scale bar: 20 μm.

**D.** H&E staining of proximal tibia and femur, and PAS staining of lung in normal and B16F10 metastasis mice, with or without daily 10 mg/kg Roxadustat (n=5 per group).

**E.** H&E staining of cortical bones in normal and B16F10 metastasis mice, with or without daily 10 mg/kg Roxadustat. Quantification of filled, dying, and empty lacunae (n =5 per group). White arrows: normal osteocytes; green arrows: dying osteocytes; red arrows: dead osteocytes. Scale bars: 20 μm.

**F.** mRNA expression of Dmp1, Dkk1, Sclerostin, and Phex in long bone (without marrow) of normal and B16F10 metastasis mice, with or without daily 10 mg/kg Roxadustat (n=4 per group).

**G.** mRNA expression of Bax, Caspase 3, and Caspase 8 in long bone tissue from B16F10 metastasis mice treated or not with 10 mg/kg Roxadustat (n≥10 per group).

Statistical significance was determined by a 2-tailed Student’s t-test (B, G) or one-way ANOVA (C, D, E, F).

**Supplementary Figure 8**

**A.** Heatmap of differentially expressed genes (DEGs) in lysosome pathways B16F10 metastasis mice with or without daily 10 mg/kg Roxadustat.

**B.** TRAP staining and quantification of osteoclast surface/bone surface (Oc.S/BS), osteoclast number/tissue area (N.Oc/T.Ar), and osteoclast number/bone perimeter (N.Oc/B.Pm.) in normal and B16F10 metastasis mice, with or without daily 10 mg/kg Roxadustat (n≥5 per group).

Statistical significance was assessed by one-way ANOVA.
