# Supplementary material for: Melanoma bone metastasis-induced osteocyte ferroptosis via the HIF1α-HMOX1 axis
Source: Bone Res. 2025 Jan 16;13:9. doi: 10.1038/s41413-024-00384-y (PMC11735842; doi:10.1038/s41413-024-00384-y)
Supplement: Supplementary file 8 — Supplementary Figure 8 [file 41413_2024_384_MOESM8_ESM.pdf]

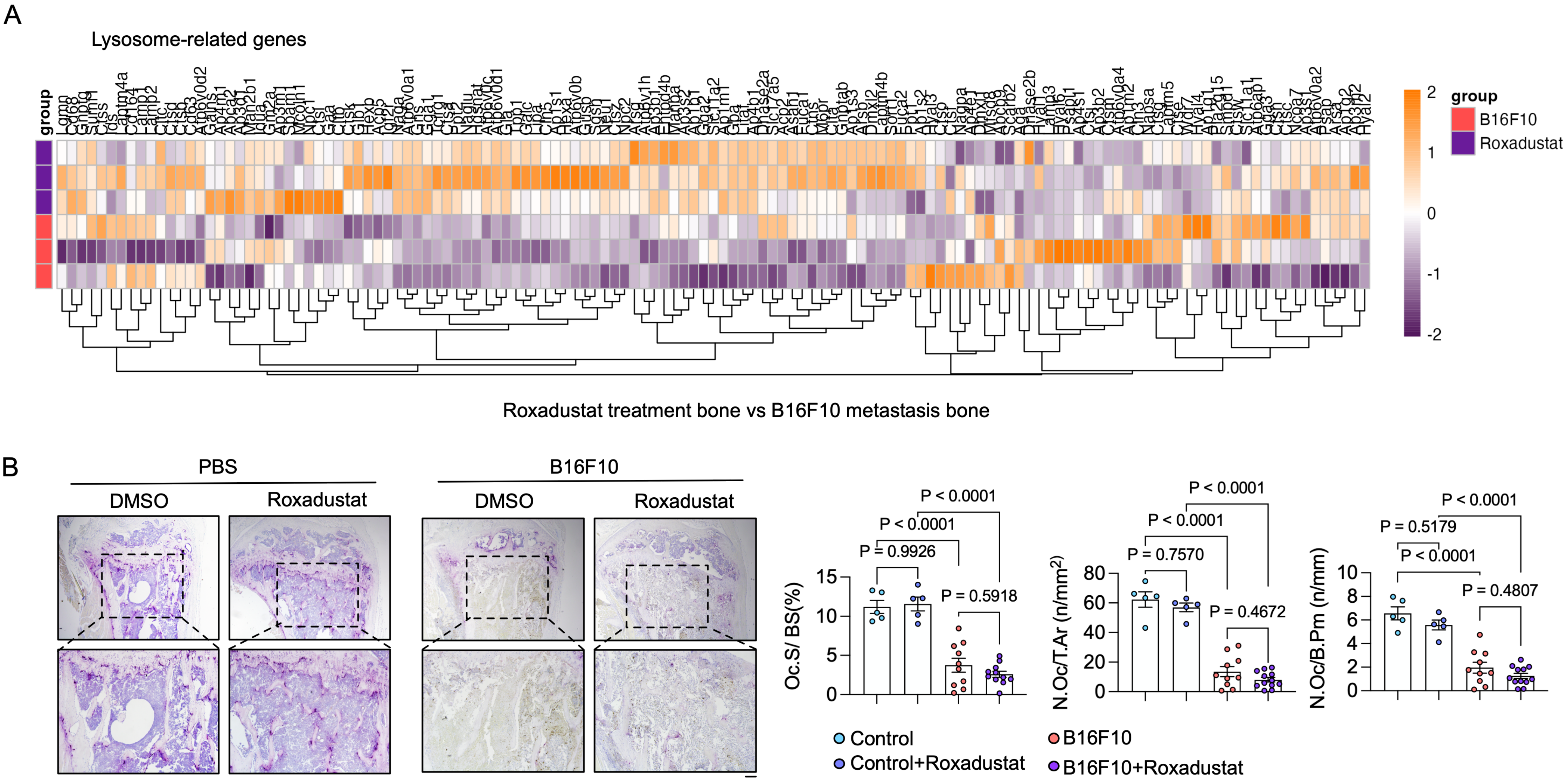
A
Lysosome-related genes
group
group
B16F10
Roxadustat
2
1
0
-1
-2
Roxadustat treatment bone vs B16F10 metastasis bone
B
PBS
B16F10
DMSO
Roxadustat
DMSO
Roxadustat
Oc.S/ BS(%)
15
10
5
0
P < 0.0001
P < 0.0001
P = 0.9926
P = 0.5918
N.Oc/T.Ar (n/mm2)
80
60
40
20
0
P < 0.0001
P < 0.0001
P = 0.7570
P = 0.4672
N.Oc/B.Pm (n/mm)
10
8
6
4
2
0
P < 0.0001
P = 0.5179
P < 0.0001
P = 0.4807
Control
Control+Roxadustat
B16F10
B16F10+Roxadustat
